# Comparative Analysis of the Daily Liver Metabolomics of Asian Particolored Bat

**DOI:** 10.1002/ece3.71666

**Published:** 2025-06-27

**Authors:** Yujia Chu, Hui Wang, TianHui Wang, Jingjing Li, Lei Feng, Hui Wu, Tinglei Jiang, Jiang Feng

**Affiliations:** ^1^ College of Life Science Jilin Agricultural University Changchun China; ^2^ Jilin Provincial International Cooperation Key Laboratory for Biological Control of Agricultural Pests Changchun China; ^3^ Jilin Provincial Key Laboratory of Animal Resource Conservation and Utilization Northeast Normal University Changchun China

**Keywords:** bats, circadian rhythms, liver, metabolites

## Abstract

Mammals rely on an intricate network of circadian clocks to regulate daily metabolic and physiological processes throughout the body. The liver, as a key peripheral organ, plays a crucial role in coordinating circadian rhythms and metabolic regulation. Bats, the only mammals capable of true flight, present unique nocturnal behaviors, which have garnered significant research interest. However, the daily metabolic fluctuations in bat liver and the underlying mechanisms of circadian regulation remain largely unexplored. To elucidate the metabolic foundations and rhythmic patterns in bat liver, a comparative LC–MS analysis was conducted to characterize the type, composition, and abundance of metabolites in the liver of Asian particolored bats at four distinct time points throughout a single day. This analysis identified a total of 7211 metabolites, with approximately 2.0% exhibiting significant rhythmicity, though with varying rhythmic patterns. Integrating these findings with RNA‐Seq data revealed that differentially expressed genes and differentially accumulated metabolites were significantly enriched in two key pathways: the protein digestion and absorption pathway and the glutathione metabolism pathway. Notably, our results suggest that L‐glutamate and L‐tryptophan may play pivotal roles in the metabolic regulation of hepatic circadian rhythms, as indicated by metabolomics analysis. This study provides a systematic examination of the cyclic variations in metabolites within the bat liver, uncovering the physiological and biochemical processes involved and thereby enhancing our understanding of the molecular mechanisms governing circadian rhythms in bats.

## Graphical abstract

This study elucidated the composition of liver metabolites and clarified the rhythmic patterns of critical metabolites and associated physiological processes in the bat liver. The circadian‐regulated metabolic patterns in bats may have significant implications for their energy strategies and survival. Antioxidant‐related physiological processes are activated both before and after bats' energy‐intensive flight behavior, preparing and protecting the body from oxidative stress.

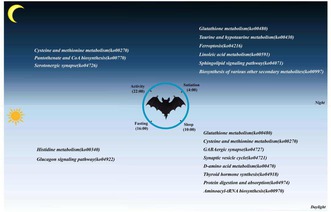

## 1 Introduction

The circadian clock is a highly conserved endogenous timing system that enables organisms, from bacteria to mammals, to synchronize their physiological functions with Earth's environmental cycles (Panda 2016; Pittendrigh 1993; Wang et al. 2022). In mammals, this clock operates across nearly all tissues and cells (Balsalobre et al. 1998; Yoo et al. 2004), consisting of a central clock located in the suprachiasmatic nuclei (SCN) of the hypothalamus, along with peripheral clocks dispersed throughout various tissues (Dibner et al. 2010; Mohawk et al. 2012), which are influenced by multiple zeitgebers such as light and feeding cues (Gnocchi et al. 2015; Roenneberg et al. 2013; Stokkan et al. 2001). While the SCN serves as the master regulator of circadian rhythms, peripheral organs such as the liver maintain their own autonomous rhythms, even in the absence of SCN input. This autonomy suggests that peripheral tissues operate independent cellular clocks (Kent et al. 2022; Koronowski et al. 2019).

The liver, as a critical metabolic organ, plays a central role in regulating various rhythmic metabolic pathways (Heymann and Tacke 2016). Previous studies have demonstrated that the expression of metabolism‐related genes in the liver exhibits pronounced rhythmicity (Eckel‐Mahan et al. 2013; Hatori et al. 2012), playing a critical role in regulating various metabolic processes such as lipid, glucose, and protein metabolism (Reinke and Asher 2016). Kent et al. discovered that the hepatic clock can independently drive specific aspects of metabolism and is influenced by behavioral zeitgebers such as the sleep–wake cycle and feeding‐fasting cycle (Koronowski et al. 2019; Skene et al. 2018). Moreover, many of liver metabolites, including melatonin, glucose, hemoglobin, and bilirubin, display pronounced circadian rhythmicity (Dyar et al. 2018; Sanchez et al. 2018; Sulli et al. 2018; Wang et al. 2019). These metabolites not only govern local metabolic processes but also influence the functioning of other peripheral clocks, emphasizing the liver's hierarchical regulatory role in coordinating circadian rhythms systemically (Johnston et al. 2016). Thus, studying the liver's circadian clock is of great importance, given its pivotal role in controlling metabolic processes and influencing peripheral tissues.

Despite the extensive body of research on circadian rhythms in model organisms like mice and rats (Eckel‐Mahan et al. 2013), there is a notable lack of studies on circadian clocks in wild animals under natural conditions. Bats, as the only true flying nocturnal mammals, provide a unique opportunity for circadian research. With over 1400 species, bats are one of the most diverse groups of mammals, inhabiting a wide range of geographical areas and exhibiting specialized adaptations such as echolocation, flight, immune responses, and extended lifespans (Burgin et al. 2018). These adaptations to a nocturnal lifestyle make bats particularly intriguing for the study of circadian rhythms.

Bats also play crucial ecological roles, contributing to pollination, seed dispersal, and pest control (Ferreira et al. 2024). However, most research on bats has traditionally focused on their behavioral and ecological aspects, such as echolocation, viral transmission, and population distribution (Jebb et al. 2020; Li et al. 2021; Liu et al. 2022), with limited investigation into their molecular circadian mechanisms. Uncovering the rhythmic changes in key energy metabolism processes, such as lipid and glucose metabolism, in the bat liver can help elucidate how bats balance energy expenditure and storage under the regulation of the circadian rhythm, then providing insights into the adaptive evolution of nocturnal mammals and the temporal differentiation of ecological niches.

The advent of molecular techniques such as transcriptomics and metabolomics has significantly advanced our ability to study circadian rhythms in wildlife species, including bats. Metabolomic analyses, in particular, allow researchers to explore the rhythmic changes in metabolites linked to circadian cycles, offering key insights into the regulation of metabolic processes within circadian frameworks. In recent years, metabolomics has emerged as a powerful tool for investigating circadian rhythms, particularly in nonmodel organisms such as bats. By capturing and analyzing rhythmic fluctuations in metabolites over a 24‐h cycle, metabolomics provides unique insights into the complex interactions between metabolic processes and circadian regulation (Malik et al. 2020). Metabolomics enables a comprehensive examination of how liver metabolites are linked to the circadian clock, bridging molecular rhythms with physiological functions. Given the ecological and physiological complexity of bats, metabolomics offers insights that are difficult to achieve with traditional circadian studies alone.

In this study, we chose the Asian particolored bat ( *Vespertilio sinensis* ), a widely distributed and observable species, to elucidate the molecular mechanisms underlying the liver's circadian rhythms. We focused on four distinct physiological states: satiation, sleep, fasting, and activity, to capture key phases of the bat's 24‐h circadian cycle, thereby reflecting the diverse metabolic demands, including satiation–fasting and sleep–activity cycles. Using liquid chromatography‐mass spectrometry (LC–MS), we compared liver metabolite levels across different physiological states over a 24‐h period. Additionally, transcriptomic data were integrated to address three core questions: (1) How do the composition and levels of liver metabolites vary across different physiological states in Asian particolored bats, and what metabolic processes are involved in these variations? (2) What rhythmic patterns can be observed in the liver metabolites of Asian particolored bats over a 24‐h period, and how are these rhythms related to the liver's circadian clock and associated physiological processes? (3) How might these rhythmic metabolites contribute to the regulation of the peripheral circadian clock? This study explores the molecular mechanisms of circadian rhythms in the liver of the Asian particolored bat, providing new insights into how nocturnal mammals adapt metabolically to their environment. By linking metabolomics with circadian biology, it broadens our understanding of circadian regulation in wild species and contributes to conservation efforts for nocturnal animals.

## 2 Methods and Materials

### 2.1 Sample Collection

**FIGURE 1:**
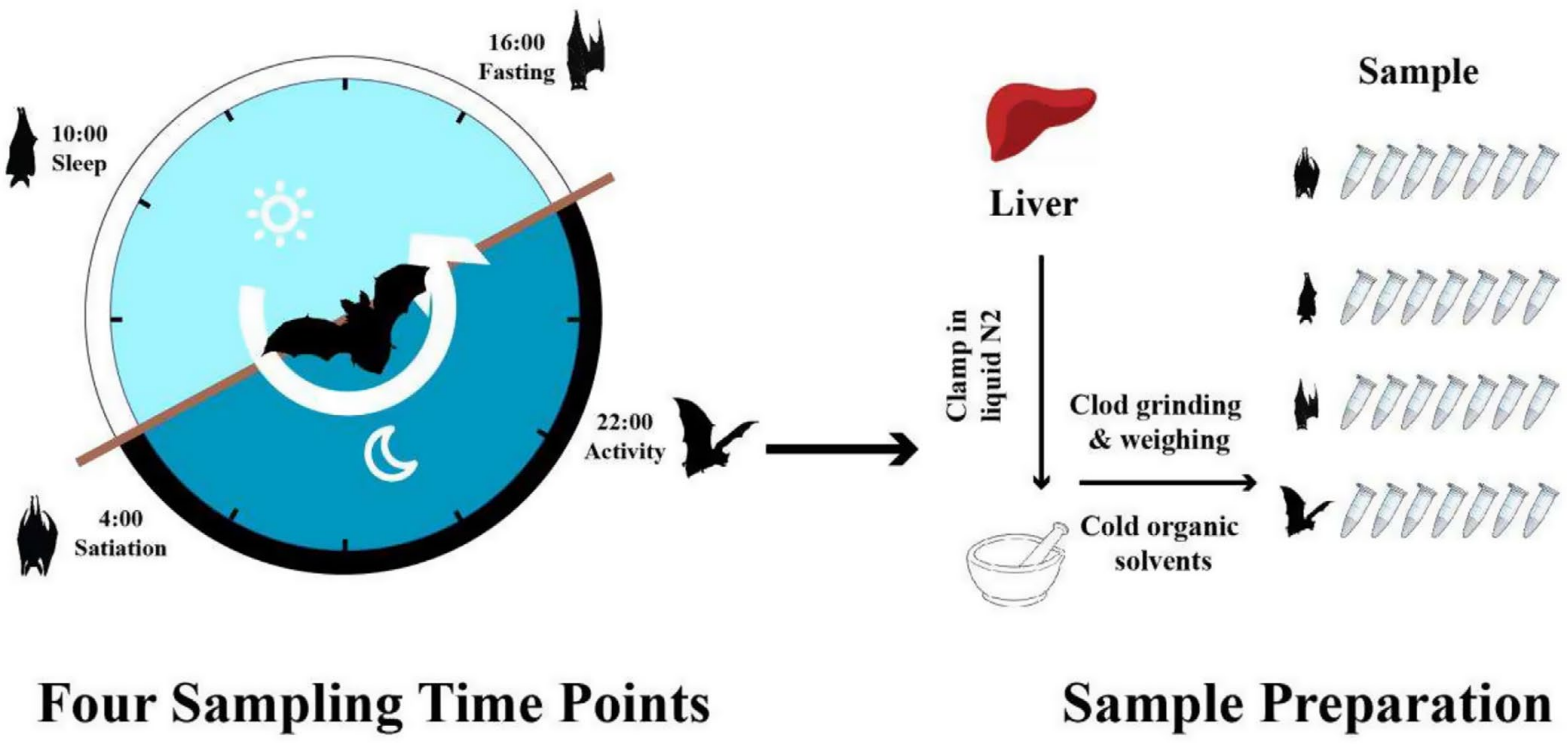
Schematic flow of experimental design.

This study was conducted in a colony of Asian particolored bats located in Acheng District, Harbin, Heilongjiang Province (45°32′55″ N, 127°32′59″ E) from July to August 2020. Observations of this bat group were carried out over a week to determine their daily activity pattern across a 24‐h cycle (Figure 1). Based on these observations, four representative activity states were selected for sampling: 4:00 (satiation), 10:00 (sleep), 16:00 (fasting), and 22:00 (activity). All samples were collected within a single day at these four time points. During the satiation, sleep, and fasting states, bats were captured using hand‐held nets, while for the active state, mist nets were employed. To minimize the effects of reproductive status and to avoid sex‐related differences in the data, only nonpregnant, nonlactating female bats were selected. For each physiological state, six adult female bats were collected to serve as biological replicates, and a total of liver tissues from 24 bat individuals (with an average weight of approximately 20 g) were collected for this study. To minimize suffering, each captured bat was sacrificed by decapitation at one of the four predetermined time points throughout the day. All animal experimental procedures were approved by the National Animal Research Authority of Northeast Normal University, China (approval number: Nenu‐20080416), and the Forestry Bureau of Jilin Province, China (approval number: [2006] 178). All efforts were made to minimize the suffering of the animals. We confirm that all methods were performed in accordance with the relevant guidelines and regulations mentioned above. We declare that this study is reported in accordance with ARRIVE guidelines.

Regarding liver sampling, due to the small body size of bats and the need to maintain metabolic integrity, in situ perfusion with saline was not carried out. Immediately after excision, liver tissues were gently rinsed with ice‐cold phosphate‐buffered saline (PBS). This aimed to remove surface blood as effectively as possible, following the standard practice in small mammalian metabolomics studies (Eckel‐Mahan et al. 2013; Hatori et al. 2012). After rinsing, liver tissues were carefully excised, placed on ice for rapid handling, and immediately transferred to RNase‐free freeze tubes. The samples were then snap‐frozen in liquid nitrogen and stored overnight. Subsequent metabolite extraction and analysis were performed on these samples.

### 2.2 Sample Preparation for LC–MS Analysis

For each of the 24 bat liver samples, 20 μL of L‐2‐chlorophenyl alanine (0.3 mg/mL) and Lys‐QPC17:0 (0.01 mg/mL), both prepared in methanol, were added, followed by 400 μL of methanol–water solution (4:1, v/v). The mixture was vortexed thoroughly, then subjected to ultrasonication for 10 min in an ice‐water bath, and subsequently left at −20°C for 30 min. Afterwards, the sample was centrifuged at 13,000 rpm for 10 min at 4°C, and 300 μL of the supernatant was collected and evaporated to dryness. The dried extract was reconstituted in 200 μL of methanol–water solution (1:4, v/v), vortexed for 30 s, sonicated for 3 min, and allowed to stand at −20°C for 2 h. The sample was then centrifuged again at 13,000 rpm for 10 min at 4°C, and 150 μL of the supernatant was collected. After filtering through a 0.22 μm organic phase pinhole filter, the sample was transferred to an LC injection vial for LC–MS analysis. Quality control (QC) samples were prepared by pooling equal volumes of extracts from all samples, ensuring that each QC sample volume matched that of individual samples.

### 2.3 LC–MS Analysis and Functional Annotation

Liquid chromatography‐mass spectrometry analysis was conducted using a LC–MS system composed of a Nexera UPLC ultra‐high performance liquid chromatography coupled with a QE high‐resolution mass spectrometer. Samples were analyzed using an ACQUITY UPLC HSS T3 column (100 mm × 2.1 mm, 1.8 μm) with gradient elution. The mobile phase comprised water with 0.1% formic acid for Channel A and acetonitrile/methanol with 0.1% formic acid for Channel B. The LC parameters were as follows: flow rate of 0.35 mL/min, column temperature of 45°C, and injection volume of 2 μL. Detection was performed using an electrospray ionization (ESI) probe with positive and negative polarity switching. The MS parameters included a spray voltage of 3.5 kV for positive mode and 3.0 kV for negative mode, with a probe temperature of 320°C and a full scan range from 125 to 1000 m/z. To enhance calibration stability, lock‐mass correction was applied in each analytical run using prevalent low‐mass contaminants. The parallel reaction monitoring (PRM) parameters included a resolution of 17,500, with collision energy independently set for high‐energy collisional dissociation (HCD) mode (Figures S1–S4).

Baseline filtering, compound identification, integration, retention time correction, peak alignment, and data normalization were performed using Progenesis QI v2.3 software (Zhang et al. 2016) (Nonlinear Dynamics, Newcastle, UK). Compound identification relied on precise mass measurements, secondary fragmentation patterns, and isotopic distributions. Identification and relative quantification were conducted using the Human Metabolome Database (Wishart et al. 2018) (HMDB), LipidMaps (Cotter et al. 2006) (v2.3), METLIN (Smith et al. 2005), and a custom‐built library database.

### 2.4 Detection of Differentially Accumulated Metabolites and Functional Enrichment Analysis

To assess the distribution across samples and ensure the stability of the overall analysis, we conducted a principal component analysis (PCA) with a 95% confidence interval. Subsequently, partial least squares discrimination analysis (PLS‐DA) and orthogonal partial least squares discriminant analysis (OPLS‐DA) (with 200 permutations) were employed to discern metabolite differences between groups. Using LC–MS, we analyzed 24 liver tissue samples from Asian particolored bats to extract metabolites. Based on quality control results, one outlier sample (Satiation‐6) was excluded from further data analysis (Figures S5–S9).

To elucidate diurnal changes in bat liver metabolites, sequential comparisons were conducted between metabolites at two adjacent time points: satiation vs. sleep, sleep vs. fasting, fasting vs. activity, and activity vs. satiation. Additionally, comparisons were made between two sets of contrasting physiological states: satiation vs. fasting and sleep vs. activity. This approach resulted in six differential metabolite comparison groups. We then combined these six sets of differentially accumulated metabolites (DAMs) to create a comprehensive list of metabolites that exhibited significant changes across any of the comparisons. Finally, a heatmap was constructed to visually represent the abundance variations of these metabolites across the four time points, highlighting the differences and dynamic performance of these metabolites over time.

The criteria for identifying DAMs in each comparison group were a variable importance of projection (VIP) score > 1, and a *p* value < 0.05. DAMs were subsequently subjected to enrichment analysis using the KEGG database (http://www.kegg.jp) and MetaboAnalyst (http://www.metaboanalyst.ca/), with a significance threshold of *p* value < 0.05 for identifying pathways significantly enriched by DAMs.

### 2.5 Rhythmic Metabolite Screening and Functional Enrichment Analysis

To elucidate the rhythmic changes of liver metabolites in Asian particolored bats over a 24‐h period, rhythmic analyses were conducted separately for all metabolites and DAMs, respectively. Rhythmic analysis was performed using DiscoRhythm (Carlucci et al. 2019) software, applying the relative abundance of all metabolites and DAMs across different physiological states. Significant rhythmic changes were identified with a threshold of *p* value < 0.05. Functional classification of rhythmic metabolites was achieved by mapping to KEGG pathway databases. This analysis aimed to compare the functional classification and enrichment differences between all metabolites and DAMs with cyclic patterns. Through this approach, we sought to identify which functions play a dominant role in the regulatory network of the liver's cyclic variation.

### 2.6 Integrated RNA‐Seq and Metabolomics Analysis

In a prior liver transcriptomics study on Asian particolored bats, gene expression data were obtained at the same time points as this research, identifying differentially expressed genes (DEGs) and enriched pathways across four physiological states (Chu et al. 2022). The gene expression data from Chu et al. (2022) were indeed derived from the same batch of liver samples used in this study. After homogenization using liquid nitrogen, each liver sample was divided into two portions: one for RNA sequencing (used in Chu et al. 2022) and the other for metabolomic analysis (reported herein). In this study, we conducted a correlation analysis integrating transcriptomic and metabolomic data to uncover the physiological processes and molecular mechanisms underlying circadian rhythm regulation in Asian particolored bats at both transcriptional and metabolic levels.

First, we compared the enrichment analysis results of DEGs across each state with those of the DAMs to identify pathways significantly enriched in both genes and metabolites. Furthermore, functional enrichment analysis of DEGs and DAMs was conducted jointly using MetaboAnalyst (Pang et al. 2021), with a significance threshold of *p* value < 0.05. From this, we constructed a gene–metabolite correlation network to identify key pathways, genes, and metabolites. This integrated network of genes and metabolites was created using the Metscape plugin in Cytoscape software (Reimand et al. 2019). The transcriptomic data for this study were derived from the same liver tissues used here, with RNA‐Seq data obtained from our previous study (Chu et al. 2022).

## 3 Results

### 3.1 Composition and Classification of Liver Metabolites in Asian Particolored Bats

A total of 16,359 metabolites were identified through LC–MS analysis of metabolite extracts from 24 liver tissue samples taken from Asian particolored bats. Of these, 7211 metabolites were annotated within comprehensive databases: 2241 metabolites were identified in the HMDB database, 891 in the LipidMaps database, and 4079 in the METLIN and self‐built databases (Table 1 and Table S1). The 7211 annotated metabolites were classified into 20 superclasses, 195 classes, and 357 subclasses. At the superclass level, the top five metabolite categories included lipids and lipid‐like molecules, organic acids and derivatives, organic heterocyclic compounds, organic oxygen compounds, and benzenoids (Table S1).

**TABLE 1:** Basic information of the liver metabolomics of Asian particolored bats.

Identification	Metabolites	Anno. metabolites	Superclass	Class	Subclass
Positive ion	8564	4031	2340	2338	2143
Negative ion	7795	3180	1806	1803	1643
Total	16,359	7211	4146	4141	3786

**FIGURE 2:**
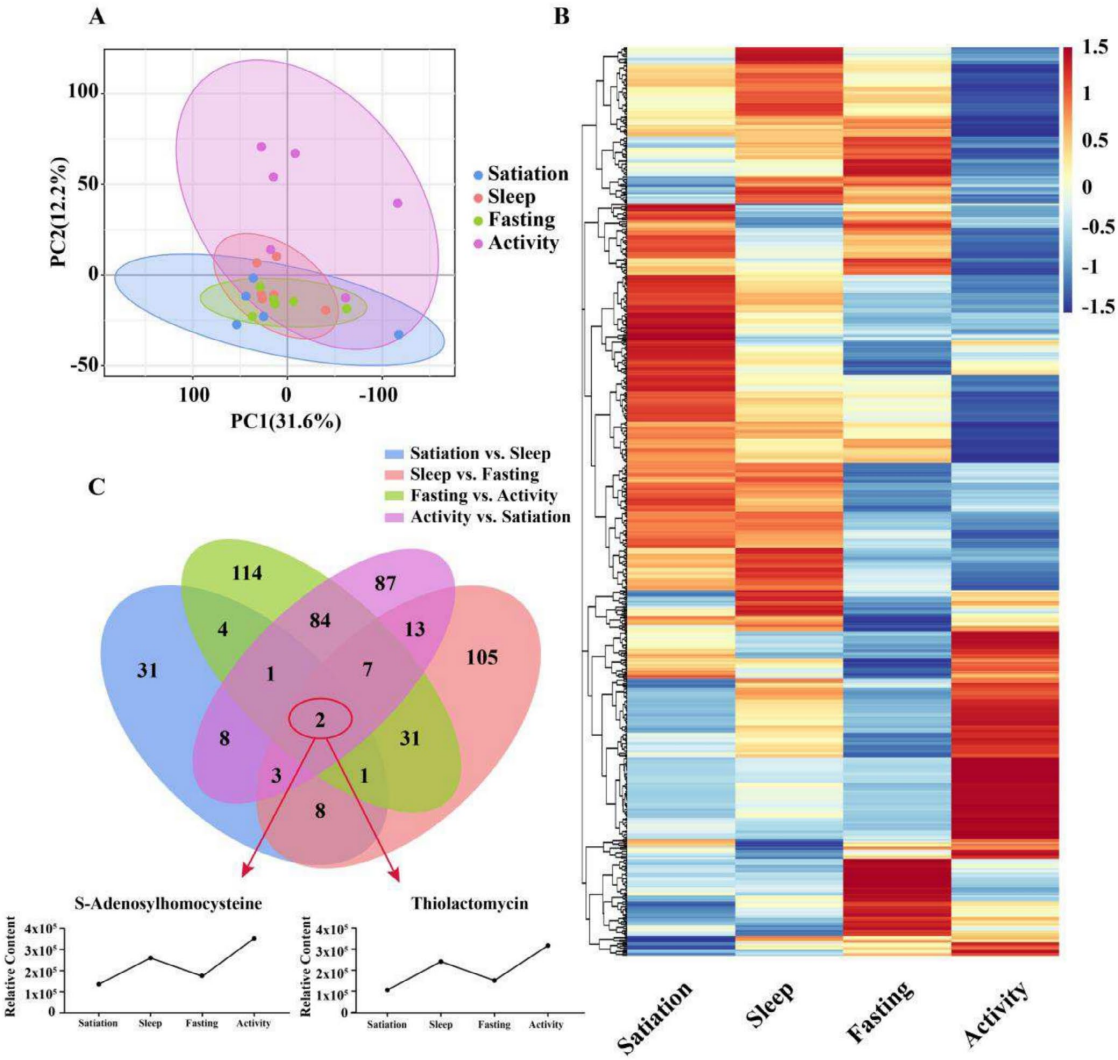
(A) Principal component analysis (PCA) of the metabolomics of four states. (B) Heatmaps of 599 DAMs from six pairwise comparisons across four time points corresponding four physiological states. (C) Venn Diagram of DAMs from four pairwise comparisons involving every two adjacent time points.

The PCA results indicated that the satiation‐6 sample was an outlier (Figure S6). After removing satiation‐6, the PCA analysis showed slightly reproducibility among samples within the same group (Figure 2A). Additionally, the PLS‐DA and OPLS‐DA analyses demonstrated further separation of samples across the four stages (Figures S7–S9). The “reproducibility” here refers to the intragroup consistency in directional metabolic trends, rather than absolute numerical similarity. Figure 2A shows that the active state accounts for the majority of the variation in metabolite accumulation observed in other states along the first principal component (PC1) and a significant amount along the second principal component (PC2), suggesting that the metabolite composition during active states is highly variable and closely resembles that of other states.

### 3.2 Results of Differentially Accumulated Metabolites and Functional Enrichment Analysis Across Four Physiological States

Metabolites obtained from the four physiological states were analyzed comparatively, yielding six differential comparison groups: satiation vs. sleep, sleep vs. fasting, fasting vs. activity, activity vs. satiation, satiation vs. fasting, and sleep vs. activity. Across these comparisons, 58, 170, 244, 205, 123, and 243 DAMs were identified, respectively, totaling 599 unique DAMs (Table S2). These DAMs were primarily classified into 12 major superclasses, with lipids and lipid‐like molecules, organic acids and derivatives, organic heterocyclic compounds, organic oxygen compounds, phenylpropanoids and polyketides, and nucleosides, nucleotides, and analogs comprising significant proportions (Table S2).

The comparison with the fewest DAMs was satiation vs. sleep, while the highest numbers were found in the fasting vs. activity and sleep vs. activity groups, with carboxylic acids and derivatives being predominant. Notably, in the satiation vs. fasting group, organic heterocyclic compounds showed significant differences compared to other groups. Further analysis of the DAMs indicated that carboxylic acids and derivatives, organic oxygen compounds, fatty acyl groups, glycerophospholipids, purine nucleosides, flavonoids, and imidazopyridines were abundantly represented. In total, 599 DAMs were identified across the six differential comparison groups (Figure 2B). Among these, S‐adenosylhomocysteine and thiolactomycin exhibited significant variation across successive physiological states (Figure 2C).

**FIGURE 3:**
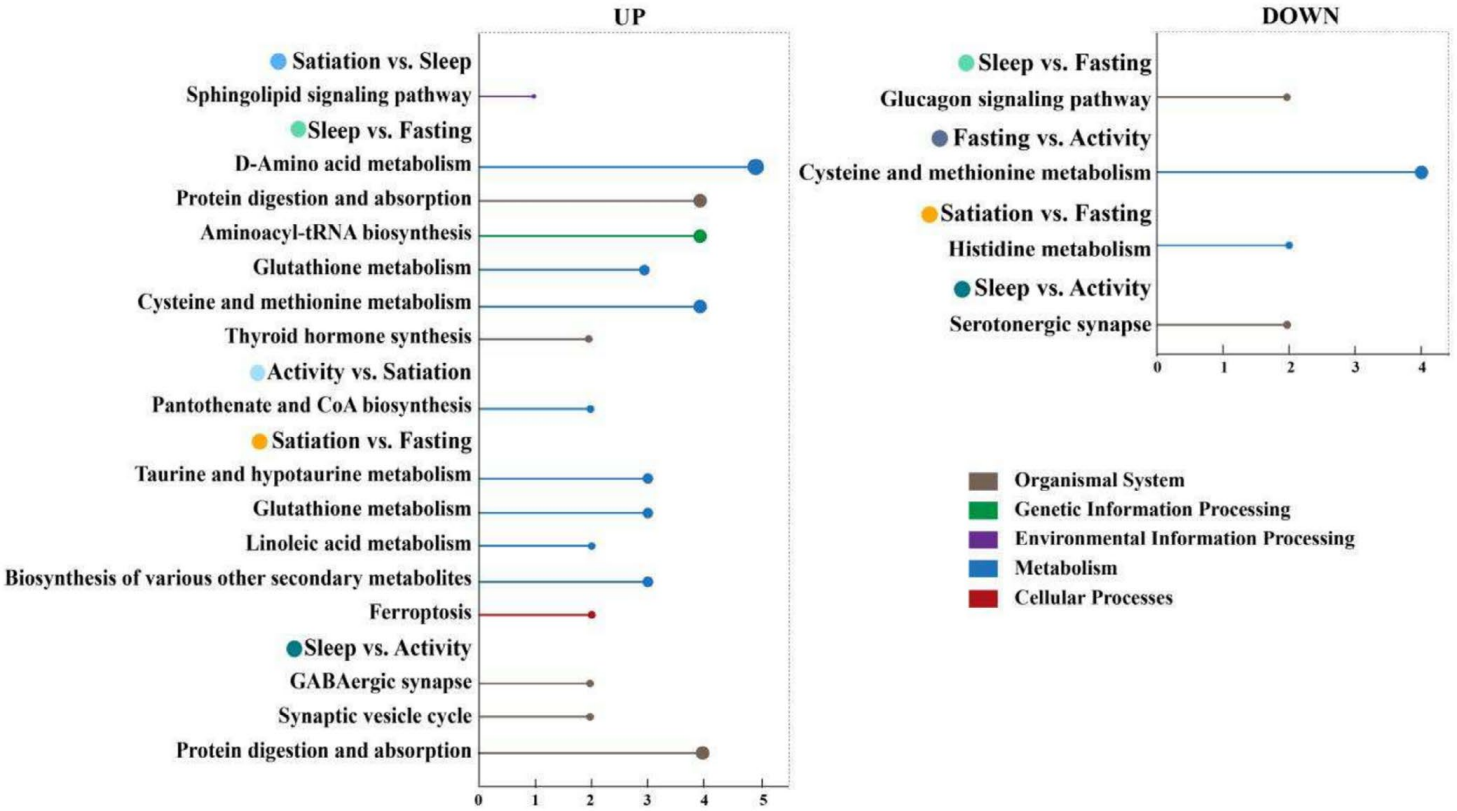
KEGG pathways significantly enriched by DAMs detected from six pairwise comparisons (*p* value < 0.05). Pathways enriched by higher abundant metabolites detected in the former state than in the latter of one comparison are shown in the left, and pathways enriched by higher abundant metabolites detected in the latter state are shown in the right.

The functional enrichment analysis of DAMs across the six comparison groups is summarized in Table 2 and Table S3. In the satiation vs. sleep group, DAMs with higher abundance in the satiation state were significantly enriched in pathways such as the sphingolipid signaling pathway (ko04071), while no pathways were significantly enriched in the sleep state. In the sleep vs. fasting group, DAMs more abundant in the sleep state were significantly enriched in pathways including D‐amino acid metabolism (ko00470), protein digestion and absorption (ko04974), aminoacyl‐tRNA biosynthesis (ko00970), cysteine and methionine metabolism (ko00270), glutathione metabolism (ko00480), and thyroid hormone synthesis (ko04918). Conversely, DAMs with higher levels in the fasting state were significantly enriched in the glucagon signaling pathway (ko04922) (Figure 3). For the fasting vs. activity group, no DAMs were significantly enriched in pathways during the fasting state. However, DAMs more abundant in the activity state were significantly enriched in the cysteine and methionine metabolism pathway (ko00270). In the activity vs. satiation group, DAMs higher in the activity state were enriched in pathways such as pantothenate and CoA biosynthesis (ko00770), whereas no pathways were significantly enriched in the satiation state. In the satiation vs. fasting group, DAMs with higher levels in the satiation state were significantly enriched in pathways such as taurine and hypotaurine metabolism (ko00430), glutathione metabolism (ko00480), biosynthesis of various other secondary metabolites (ko00997), linoleic acid metabolism (ko00591), and ferroptosis (ko04216). DAMs more abundant in the fasting state showed significant enrichment in the histidine metabolism pathway (ko00340) (Figure 3). Lastly, in the sleep vs. activity group, DAMs with higher abundance in the sleep state were enriched in pathways such as protein digestion and absorption (ko04974), GABAergic synapse (ko04727), and synaptic vesicle cycle (ko04721), while DAMs more abundant in the activity state were significantly enriched in the serotonergic synapse pathway (ko04726) (Figure 3).

**TABLE 2:** Number of upregulated and downregulated DAMs detected in six pairwise comparison groups and the number of KEGG pathways that were significantly enriched by DAMs.

	Differentially accumulated metabolites (DAMs)	KEGG pathway
	Up/down	Up/down
Satiation vs. sleep	21//37	1/0
Sleep vs. fasting	114/56	8/2
Fasting vs. activity	119/125	0/1
Activity vs. satiation	147/58	1/0
Satiation vs. fasting	77/46	6/1
Sleep vs. activity	195/48	4/2

*Note:* Metabolites with higher abundant level detected in the former state than in the latter of one comparison were defined as upregulated, and vice versa for a downregulated metabolite.

**FIGURE 4:**
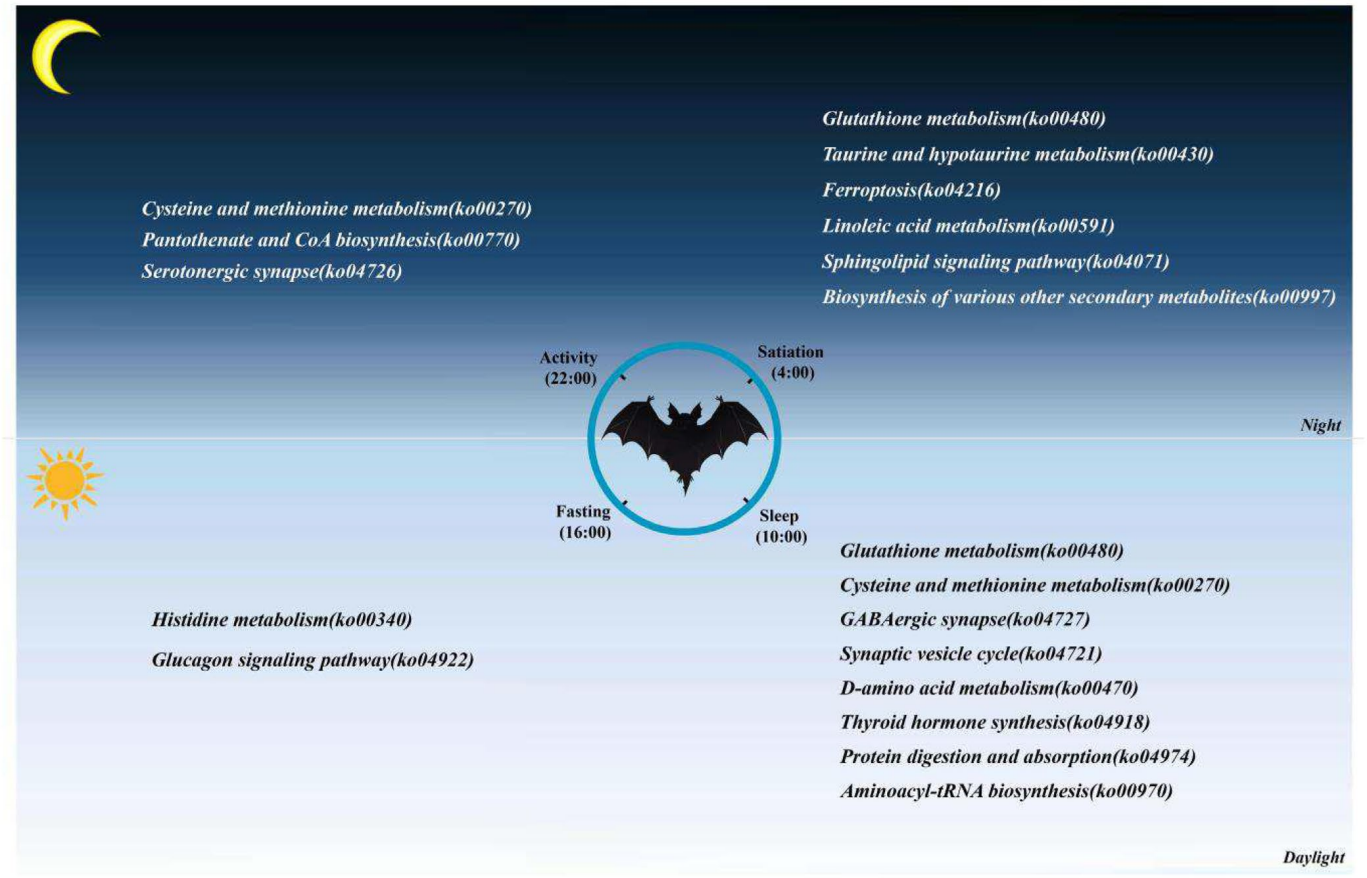
Summary of the more active physiological processes for each state in the bat liver.

We compiled a summary figure to illustrate pathways significantly enriched with high‐abundance DAMs in each physiological state (Figure 4). In the satiation state, metabolites with high abundance were significantly enriched in six pathways: glutathione metabolism (ko00480), taurine and hypotaurine metabolism (ko00430), ferroptosis (ko04216), linoleic acid metabolism (ko00591), sphingolipid signaling (ko04071), and the biosynthesis of various secondary metabolites (ko00997). For the sleep state, high‐abundance metabolites were significantly enriched in eight pathways: glutathione metabolism (ko00480), cysteine and methionine metabolism (ko00270), GABAergic synapse (ko04727), synaptic vesicle cycle (ko04721), D‐amino acid metabolism (ko00470), thyroid hormone synthesis (ko04918), protein digestion and absorption (ko04974), and aminoacyl‐tRNA biosynthesis (ko00970). In the fasting state, high‐abundance metabolites were significantly enriched in the histidine metabolism (ko00340) and glucagon signaling (ko04922) pathways. In the activity state, high‐abundance metabolites were significantly enriched in pathways including cysteine and methionine metabolism (ko00270), pantothenate and CoA biosynthesis (ko00770), and serotonergic synapse (ko04726).

In both the satiation and sleep states, DAMs were significantly enriched in the glutathione metabolism pathway (ko00480). In contrast, the cysteine and methionine metabolism pathway (ko00270) was more active in the sleep and activity states. Metabolites significantly enriched in glutathione metabolism (ko00480) and cysteine and methionine metabolism (ko00270)—including glutathione, acetyl‐CoA, S‐glutathionyl‐L‐cysteine, L‐methionine S‐oxide, and S‐nitroso‐L‐homocysteine—primarily function as antioxidants. These metabolites help neutralize free radicals and protect cells from oxidative stress‐related damage (Figure 4 and Table S3).

In the satiation state, metabolites such as S‐1‐glutamyl‐taurine and acetyl‐CoA were significantly enriched in the taurine and hypotaurine metabolism pathway (ko00430). In the sleep state, metabolites like D‐glucose 6‐phosphate were significantly enriched in the thyroid hormone synthesis pathway (ko04918). During the fasting state, metabolites such as malic acid and 2‐phospho‐D‐glycerate were significantly enriched in the glucagon signaling pathway (ko04922). In the activity state, 4′‐phosphopantetheine and glycerate were significantly enriched in the pantothenate and CoA biosynthesis pathway (ko00770). These metabolites are primarily involved in material and energy metabolism (Figure 4 and Table S3).

### 3.3 Rhythmic Metabolites and Associated Physiological Processes in the Liver of Asian Particolored Bats

**FIGURE 5:**
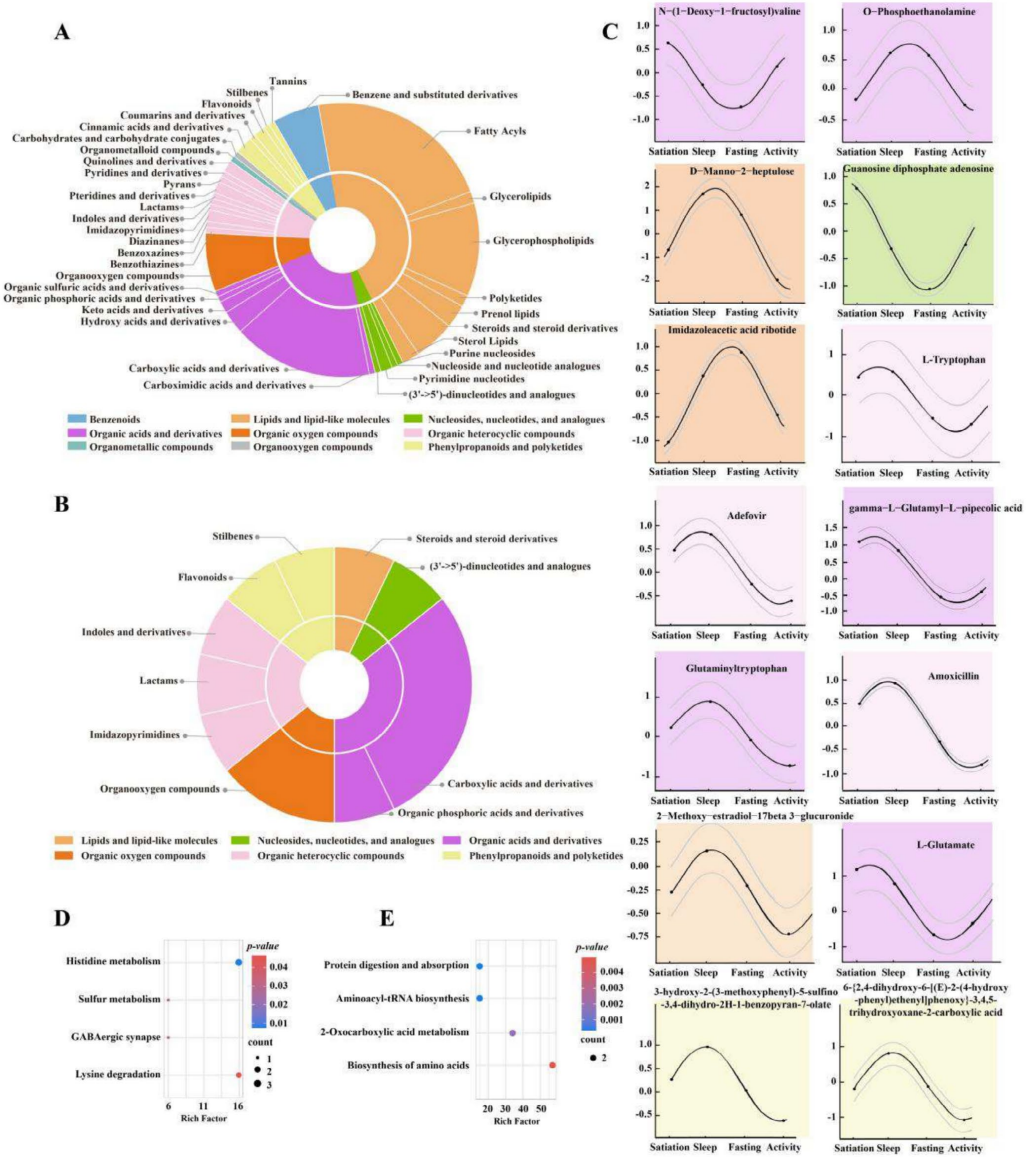
Classification of rhythmic metabolites detected from all 7211 metabolites (A) and from 599 DAMs (B), respectively. The inner doughnut represents the proportion of metabolites at superclass level, and the outer doughnut represents the proportion of metabolites at class level. (C) The daily dynamic patterns of 14 rhythmic metabolites detected from 599 DAMs. (D) and (E) are KEGG pathways significantly enriched by rhythmic metabolites from (A) and (B) group above, respectively (*p* value < 0.05).

Among the 7211 identified metabolites, 145 displayed rhythmicity (Table S4), representing 2.0% of all metabolites. These rhythmic metabolites predominantly consisted of lipids and lipid‐like molecules, organic acids and derivatives, and organic heterocyclic compounds (Figure 5A and Figure S10). They were significantly enriched in pathways such as histidine metabolism (ko00340), sulfur metabolism (ko00920), GABAergic synapse (ko04727), and lysine degradation (ko00310) (Figure 5D and Table S5).

Out of the 599 DAMs, 14 were found to be rhythmic (Table S4), comprising 2% of the DAMs. These 14 rhythmic DAMs spanned categories including organic acids and derivatives, organic heterocyclic compounds, organic oxygen compounds, nucleosides, nucleotides and analogues, phenylpropanoids and polyketides, as well as lipids and lipid‐like molecules (Figure 5B). Notable examples include N‐(1‐deoxy‐1‐fructosyl)valine, O‐phosphoethanolamine, D‐manno‐2‐heptulose, guanosine diphosphate adenosine, imidazoleacetic acid ribotide, L‐tryptophan, adefovir, gamma‐l‐glutamyl‐l‐pipecolic acid, glutaminyltryptophan, amoxicillin, 2‐methoxy‐estradiol‐17beta 3‐glucuronide, L‐glutamate, 3‐hydroxy‐2‐(3‐methoxyphenyl)‐5‐sulfino‐3,4‐dihydro‐2H‐1‐benzopyran‐7‐olate, and 6‐{2,4‐dihydroxy‐6‐[(E)‐2‐(4‐hydroxyphenyl)ethenyl]phenoxy}‐3,4,5‐trihydroxyoxane‐2‐carboxylic acid and various complex derivatives (Figure 5C). Functional enrichment analysis of these 14 rhythmic DAMs revealed significant associations with pathways such as protein digestion and absorption (ko04974), aminoacyl‐tRNA biosynthesis (ko00970), 2‐oxocarboxylic acid metabolism (ko01210), and amino acid biosynthesis (ko01230) (Figure 5E and Table S5). Notably, L‐glutamate and L‐tryptophan were involved in multiple enriched pathways, underscoring their functional importance and broad role in various cyclic physiological processes.

### 3.4 Correlation Analysis Results Between Metabolomics and Transcriptomics

Initially, we conducted enrichment analyses for DEGs and DAMs in each group separately and then compared the results. We found that in the satiation state, both DAMs and DEGs were exclusively enriched in the glutathione metabolism pathway (ko00480).

Secondly, our co‐enrichment analysis of high‐abundance DEGs and DAMs in each activity state revealed distinct patterns. In the satiation vs. fasting comparison group, both metabolites and genes were significantly enriched in the ferroptosis (ko04216), glycine, serine, and threonine metabolism (ko00260), and taurine and hypotaurine metabolism (ko00430) pathways. In the sleep vs. activity comparison group, the sleep state showed significant enrichment of metabolites and genes in pathways such as ABC transporters (ko02010), ferroptosis (ko04216), and glycolysis/gluconeogenesis (ko00010). In the activity state, both metabolites and genes were significantly enriched in the cysteine and methionine metabolism pathway (ko00270). Detailed results for other comparison groups are provided in Table S6.

**FIGURE 6:**
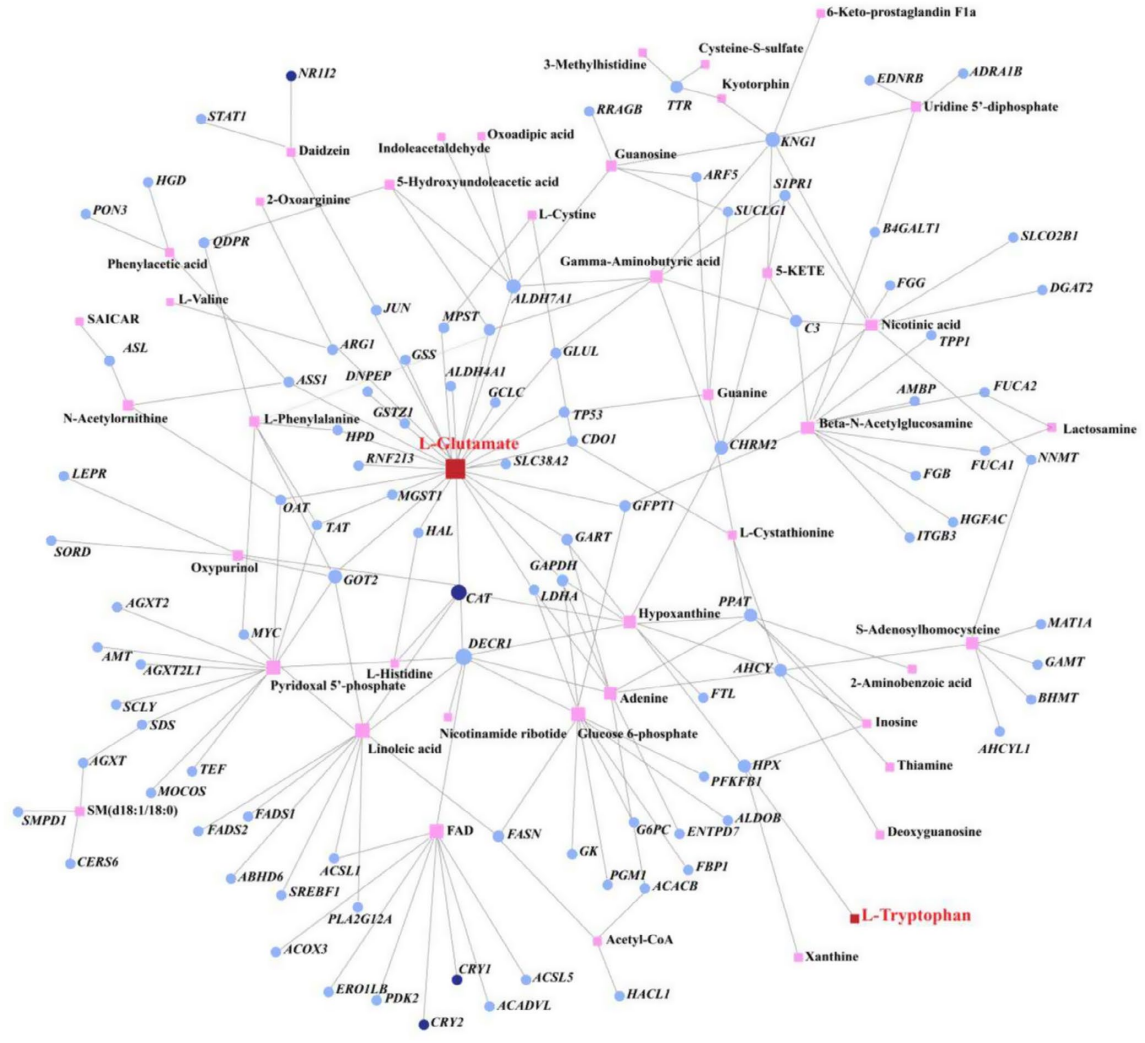
Gene–metabolite correlation network in the liver of Asian particolored bats. Squares indicate metabolites, and circles indicate genes. Lines linking genes and metabolites illustrate their correlations. Darker blue circles highlight genes associated with circadian rhythms. The size of each shape corresponds to its connectivity magnitude; larger shapes signify greater connectivity.

Finally, we co‐constructed a correlation network using all DEGs and DAMs and observed that L‐glutamate exhibited high connectivity within the network (Figure 6). These findings indicate that the glutathione metabolism pathway (ko00480) plays a prominent role in metabolic regulation at both the gene and metabolite levels, with L‐glutamate potentially participating in various rhythmic metabolic regulatory processes.

## 4 Discussion

The liver is the body's primary metabolic organ, with various metabolites involved in peripheral biological clock regulatory processes, including cyclic changes in blood glucose (Macauley et al. 2015), blood lipids (Sennels et al. 2015; van Kerkhof et al. 2015), and antioxidant functions (Joseph et al. 2024; Patel et al. 2014). This study represents the first metabolomic analysis of the liver in a wild nocturnal flying mammal. Our findings elucidated the composition of liver metabolites, clarified the rhythmic patterns of critical metabolites and associated physiological processes in the bat liver. While the use of uncorrected *p* values in our analysis may increase the risk of false positives, it also allows us to identify a broader range of potential rhythmic metabolites and pathways, thereby laying the groundwork for further investigation. We believe our findings offer a valuable foundation for future research and enrich the understanding of metabolic rhythms in wild mammals.

Understanding the circadian regulation of metabolism in bats is essential for elucidating their unique physiological adaptations and has broader ecological implications. The metabolic rhythms in bats could influence their energy strategies, survival, and interactions with the environment. For instance, the timing of metabolic processes could affect their foraging efficiency, energy expenditure during flight, and ability to cope with environmental stressors (Han et al. 2015). Additionally, comparing the metabolic rhythms of nocturnal bats with those of diurnal species can provide valuable insights into how metabolic regulation differs between activity patterns and what evolutionary advantages these differences might confer. This study provides essential reference data on flying activity, energy balance, and homeostasis in nocturnal mammals.

### 4.1 Metabolite Composition and Rhythmicity in Bat Liver and Brain

Among the metabolites detected in bat livers, the five most abundant categories were lipids and lipid‐like molecules, organic acids and derivatives, organic heterocyclic compounds, organic oxygen compounds, and benzenoids. As the central metabolic organ, the liver is vital for energy supply and storage (Li et al. 2020). Lipids and lipid‐like molecules serve as essential energy‐storage media (de Carvalho and Caramujo 2018), forming the cellular structural backbone and present in most cells (Casares et al. 2019), and constitute the largest proportion of liver metabolites. Metabolites such as organic acids and derivatives, organic heterocyclic compounds, organic oxygen compounds, and benzenoids participate in various energy metabolism processes, including the TCA cycle, glycolysis, gluconeogenesis, and other energy conversion pathways (Poggiogalle et al. 2018), forming an integrated energy and material circulation system. The metabolite composition of bat livers was similar to that of mice, but the proportion of organic heterocyclic compounds (Hancock 2021) was higher in bats than in mice (Buijink et al. 2024). This variation may be related to various factors, such as bat‐specific energy‐intensive activities such as flight and echolocation.

The metabolite composition of the liver and brain in Asian particolored bats is largely similar, with differences primarily in content. Notably, nucleosides, nucleotides, and their analogs represent a higher proportion of metabolites in the brain compared to the liver, likely reflecting the distinct functional roles of these organs. As the central control center, the brain manages processes such as motor control, perceptual integration, and autonomic nervous system regulation (Friston and Price 2001; Hernandez‐Peon and Sterman 1966) and exhibits more active physiological regulation than the liver. Recent metabolomic research indicates that approximately 23.7% of metabolites in the bat brain display rhythmic patterns (Wang et al. 2024), compared to about 2% in the liver, as observed in this study. The differences in metabolite composition and rhythmicity between the liver and brain of Asian particolored bats likely reflect their distinct functional roles. The brain's higher proportion of nucleosides, nucleotides, and their analogs, along with its greater metabolic rhythmicity, underscores its role in dynamic neural regulation and information processing. In contrast, the liver's more stable metabolic profile supports its primary function in maintaining systemic metabolic homeostasis. These findings provide valuable insights into the metabolic adaptations of bats and contribute to our understanding of how different organs regulate their metabolic activities to meet specific physiological demands.

Besides, regarding liver sampling, due to the small body size of bats (average body weight around 20 g) and the need to maintain metabolic integrity, in situ perfusion with saline was not carried out. Liver tissues were gently rinsed with ice‐cold PBS to remove surface blood as effectively as possible; however, the liver's highly vascular nature may still retain trace amounts of blood, potentially leading to the presence of blood‐derived metabolites in the samples. Blood metabolites, which often exhibit systemic circadian rhythms (e.g., glucose, insulin), might mask or dilute tissue‐specific rhythmic signals in the liver. The rhythmicity of liver metabolites is also influenced by external timing factors such as feeding cycles (Kornmann et al. 2007) and the sleep–wake cycle (Montagnese et al. 2009), resulting in differences in the rhythmic patterns and intensity between brain and liver.

### 4.2 Functional Rhythmicity of L‐Glutamate and L‐Tryptophan in Liver Metabolism

To explore metabolites that influence liver circadian rhythms, we analyzed the 14 DAMs with significant rhythmicity. Among these, L‐glutamate and L‐tryptophan displayed pronounced functional rhythmicity. Co‐enrichment analysis of the 14 rhythmic DAMs showed that L‐glutamate and L‐tryptophan were significantly enriched across multiple pathways. Our metabolomic and transcriptomic analysis of the liver in Asian particolored bats revealed that L‐glutamate and L‐tryptophan exhibit significant rhythmicity and participate in various rhythmic physiological processes. Specifically, in the satiation state, L‐glutamate was significantly enriched in ferroptosis (ko04216) and taurine and hypotaurine metabolism (ko00430). In the sleep state, L‐glutamate was enriched in pathways such as ferroptosis (ko04216), ABC transporters (ko02010), alanine, aspartate, and glutamate metabolism (ko00250), and arginine and proline metabolism (ko00330). Similarly, in the satiation state, L‐tryptophan was significantly enriched in mineral absorption (ko04978) and glycine, serine, and threonine metabolism (ko00260), while in the sleep state, it was enriched in glycine, serine, and threonine metabolism (ko00260).

In the transcription‐metabolism correlation network, only L‐glutamate and L‐tryptophan among the 14 rhythmic metabolites showed interactions with genes, with L‐glutamate also directly linked to biological clock‐related genes. As the primary excitatory amino acid in the mammalian central nervous system, L‐glutamate plays an essential role in the internal timing system (Chi‐Castaneda and Ortega 2018). Liver‐related glutamate processes can influence the sleep–wake regulatory system through modulation of glutamate release and uptake (He et al. 2019; Saper and Fuller 2017; Vaquero and Butterworth 2006). L‐tryptophan, an essential amino acid obtained from dietary intake (Trezeguet et al. 2021), undergoes 90% of its metabolic processing in the liver (Canto et al. 2015). As a precursor for serotonin and melatonin, L‐tryptophan can affect sleep regulation by influencing the rhythms of these neurochemicals (Cubero et al. 2005; Esteban et al. 2004). In both humans and mice, glutamate and tryptophan levels have been shown to exhibit significant peaks following feeding behavior, followed by a gradual decrease. This pattern is attributed to the correlation between glutamate and tryptophan levels and the enhanced amino acid absorption and protein metabolism that occur after feeding (Rigual et al. 2025). In Asian particolored bats, we observed similar rhythmic changes in glutamate and tryptophan, which varied more in response to feeding behavior. Related studies have demonstrated that dietary metabolites, such as amino acids like tryptophan, can reset circadian rhythms (Petrus et al. 2022). Therefore, we hypothesize that in Asian particolored bats, L‐glutamate and L‐tryptophan play a crucial role in the metabolic regulation of the bat liver.

### 4.3 Energy Metabolism and Metabolic Adaptation in Bats

Energy is essential for sustaining life and is achieved through the metabolic processing of various substances. The circadian‐regulated metabolic patterns in bats may have significant implications for their energy strategies and survival. Previous studies have demonstrated that organisms exhibit rhythmicity in multiple metabolic processes at both molecular and physiological levels, including cyclic variations in blood glucose (Macauley et al. 2015) and lipid levels (Sennels et al. 2015; van Kerkhof et al. 2015). Glucose and lipids are key players in these processes. Bats, in particular, have unique energy metabolic requirements due to extended flight durations, rapid energy expenditure, and specific energy storage needs (Maina 2000). Our study reveals that essential processes, such as hepatic lipid and glucose metabolism in bats, exhibit rhythmic changes closely associated with various physiological states and feeding cycles, including satiation–fasting and sleep–activity phases. These rhythms likely contribute to balancing energy consumption and storage in bats, regulated by their circadian rhythms. In the satiation state, coinciding with bats' feeding behavior, taurine, and hypotaurine metabolism—primarily involving metabolites like L‐glutamate and acetyl‐CoA (Blachier et al. 2009; Walsh et al. 2018)—facilitates nutrient conversion. During the sleep phase, the primary role of thyroid hormone synthesis is to promote hormone production, substance metabolism, and thermogenesis, supporting normal physiological functions. For instance, L‐phenylalanine is significantly enriched in pathways such as D‐amino acid metabolism, protein digestion and absorption, and aminoacyl‐tRNA biosynthesis. It aids in the conversion of energy‐storage molecules like sugars and lipids, contributing to metabolic homeostasis and the maintenance of essential physiological functions (Kuila et al. 2023; MacDonald et al. 2020). In the fasting phase, the glucagon signaling pathway primarily stimulates glycolysis, elevating blood glucose levels and generating ATP to fuel the organism's activities. During the active phase, pantothenate and CoA biosynthesis—through dephospho‐CoA and pantetheine 4′‐phosphate—promotes the metabolism of stored energy sources like lipids. This pathway generates significant amounts of acetyl‐CoA, providing energy for activities such as flight and feeding through mechanisms including the TCA cycle (Tahiliani and Beinlich 1991; von Dohren 1995).

In addition, the high variability in metabolite composition during the active state, as indicated by PCA, highlights the dynamic nature of metabolic processes during periods of heightened activity. This variability likely reflects the bats' ability to rapidly switch between different metabolic pathways to meet varying energy demands, such as flight and foraging. The significant overlap in metabolite composition between the active state and other states suggests that the metabolic landscape during active periods is highly adaptable. This variability may indicate the activation of specific metabolic pathways that are not as prominent in other states, reflecting the bats' ability to fine‐tune their metabolism in response to environmental cues and internal physiological signals. Future studies should focus on dissecting the regulatory mechanisms driving this variability, including the roles of circadian regulation, feeding behavior, and metabolic pathway interplay. Comparative analyses with other states, such as sleep and fasting, could provide further insights into the adaptive strategies employed by bats to balance energy demands and metabolic efficiency.

### 4.4 Influence of Diet on Liver Metabolic Rhythms

It has been shown that diet has a more pronounced effect on liver metabolic rhythms (Koronowski et al. 2019; Stokkan et al. 2001). Since dietary composition directly affects the availability of key metabolites and the activation of metabolic pathways, exploring how the feeding ecology of Asian particolored bats may have influenced the observed rhythmic patterns would enhance the broader significance of these findings. A high‐protein diet has been shown to inhibit lipid synthesis, enhance protein quality, increase energy expenditure, and reduce fat formation and accumulation, thereby promoting precise circadian rhythms in the liver and improving lipid metabolism and redox status (Chaumontet et al. 2015). The Asian particolored bat is a typical insectivorous bat, with its adult diet primarily consisting of protein‐rich Lepidoptera and Diptera (Li et al. 2021). Given this dietary composition, it is unsurprising that our results demonstrate pronounced rhythmicity in processes associated with protein digestion and absorption, antioxidant metabolic processes, and related energy metabolism. These findings suggest that the high‐protein diet of Asian particolored bats may play a crucial role in shaping the observed metabolic rhythms, potentially contributing to the bats' efficient energy utilization and metabolic homeostasis.

Especially, previous studies have demonstrated that the metabolic activity associated with protein digestion and absorption is most pronounced during the postprandial period, when amino acids absorbed from the intestine enter the liver via the portal vein (Daniels et al. 2023; Reynolds et al. 1994). However, in the liver of Asian particolored bats, we observed significant enrichment of DAMs in the protein digestion and absorption pathway during the sleep state. This observation challenges the conventional expectation that these processes should peak during high‐energy demand periods, such as the activity phase. We hypothesize that for nocturnal bats, the sleep phase may serve as a preparatory period, ensuring essential amino acids are available to support the energetic demands of flight and foraging. During the sleep phase, bats may accumulate amino acids through protein digestion and absorption, which can be utilized for gluconeogenesis to provide a stable energy supply during the active phase. This metabolic pattern likely aids bats in storing nutrients and repairing cells during sleep, when energy demands are low, enhancing overall physiological adaptability. Focusing on protein digestion and absorption during sleep could optimize nutrient utilization and reduce the need for immediate energy intake during the active phase. In contrast, the activity phase prioritizes energy production through glycolysis and fatty acid oxidation, explaining the lower abundance of protein digestion and absorption metabolites during this time.

In addition, blood glucose levels in mammals are regulated by both the hepatic circadian clock and dietary intake. Typically, blood glucose rises during active periods (daytime in humans, nighttime in mice) following feeding, prompting the liver to either metabolize glucose via glycolysis or store it as glycogen. Conversely, during rest or fasting phases, the liver adapts to increased energy demands by enhancing gluconeogenesis and glycogenolysis (Rui 2014). In Asian particolored bats, there were no DAMs significantly enriched in pathways associated with blood glucose changes during the satiation state, but there were DAMs significantly associated with thyroid hormone synthesis and glucagon signaling pathways during the sleep and fasting states, respectively. We hypothesize that during the sleep state, the energy requirement of the organism is reduced, but the brain is still dependent on glucose. During this period, the liver may rely on thyroid hormone synthesis to regulate glycogenolysis and gluconeogenesis to maintain blood glucose stability and continue to supply energy to the brain. As the bat awakens from sleep and enters a period of fasting, its metabolic pattern may shift from a predominantly anabolic feeding phase to a predominantly catabolic one. Fasting state, the presence of high level of accumulated metabolites that are significantly enriched in the glucagon signaling pathway and promote gluconeogenesis and fat oxidation during the transition from sleep to fasting in preparation for the more strenuous activity that follows.

### 4.5 Antioxidant Metabolic Processes and Potential Physiological Roles

The results of this study indicate that robust antioxidant processes are active in the liver of Asian particolored bats, particularly during satiation, sleep, and activity phases. More metabolites were significantly enriched in the glutathione metabolism pathway during satiation and sleep compared with the fasting state, suggesting heightened glutathione metabolism in these phases. Joint analysis further revealed that glutathione metabolism was enriched by both highly expressed genes and abundant metabolites during satiation. The primary function of glutathione metabolism in the liver is to support antioxidant processes by catalyzing reduction reactions between glutathione and NADPH through glutathione reductase, producing oxidized glutathione (Davies et al. 1983; Tunon et al. 1992). In the satiation state, acetyl‐CoA—a metabolite significantly enriched in glutathione metabolism—plays a central role. As a versatile metabolite, acetyl‐CoA is essential for various synthetic and catabolic pathways, including the synthesis of reductive carriers NADH and FADH2, and fatty acid synthesis (Walsh et al. 2018). During sleep, glutathione, another key metabolite enriched in this pathway, serves as a vital antioxidant, effectively neutralizing free radicals, reducing oxidative stress‐induced cellular damage, and promoting cellular repair and regeneration to alleviate liver burden (Biswas and Rahman 2009; Brigelius‐Flohe 1999). During satiation, bats' flight and feeding behaviors coincide, leading to substantial nutrient intake. Acetyl‐CoA, as an intermediate metabolite in various metabolic processes, is involved in the breakdown and conversion of these nutrients. In the sleep phase, following high‐intensity flight activities, reactive oxygen species (ROS) accumulate. As feeding ceases, acetyl‐CoA levels gradually decline while glutathione levels increase to counteract oxidative stress, supporting recovery and cellular maintenance.

In the present study, metabolites were significantly enriched in cysteine and methionine metabolism during both sleep and active phases, indicating that these metabolic processes remain active across these states in Asian particolored bats. Joint analysis further confirmed that cysteine and methionine metabolism are particularly active during sleep and activity. Cysteine and methionine metabolism play key roles in intracellular antioxidant defense and redox homeostasis by generating glutathione and other related compounds. Notably, glutathione and S‐glutathionyl‐L‐cysteine were significantly enriched in cysteine and methionine metabolism during the sleep phase, while L‐methionine S‐oxide was predominantly enriched in the active state. Glutathione participates in redox reactions with ROS and is converted to S‐glutathionyl‐L‐cysteine, aiding in the regulation of intracellular redox balance (Forman et al. 2009). Similarly, L‐methionine S‐oxide can protect cells from oxidative stress by neutralizing free radicals (Kniffin et al. 2014; O'Connor et al. 2022) These findings suggest that similar metabolic pathways perform antioxidant functions through distinct metabolic modes depending on the physiological state, cooperating with other functionally analogous pathways to safeguard the body against oxidative stress. During bats' flight, high‐energy consumption is coupled with substantial ROS production, necessitating robust antioxidant mechanisms to maintain physiological balance. These results also indicate that antioxidant‐related physiological processes are activated both before and after bats' energy‐intensive flight behavior, preparing and protecting the body from oxidative stress.

## 5 Conclusion

This study has provided a comprehensive analysis of the circadian regulation of metabolism in the liver of Asian particolored bats. By integrating metabolomics and transcriptomics data, we have identified key metabolic pathways and metabolites that exhibit rhythmic patterns, highlighting their potential roles in regulating the bats' metabolic rhythms. Our findings not only contribute to the understanding of metabolic adaptations in nocturnal mammals but also have broader ecological implications for their energy strategies, survival, and interactions with the environment. By understanding how metabolic rhythms align with the daily activity patterns of nocturnal mammals, we can gain insights into the evolutionary adaptations that have allowed bats to thrive in their unique ecological niches. Future research should focus on exploring the influence of diet, environmental factors, and comparisons with diurnal species to further elucidate the functional and ecological significance of the observed metabolic rhythms.

## Author Contributions

**Yujia Chu:** conceptualization (equal), data curation (equal), formal analysis (equal), investigation (equal), methodology (equal), resources (equal), visualization (equal), writing – original draft (equal), writing – review and editing (equal). **Hui Wang:** conceptualization (equal), data curation (equal), funding acquisition (equal), project administration (equal), supervision (equal), writing – review and editing (equal). **TianHui Wang:** investigation (equal). **Jingjing Li:** investigation (equal), methodology (equal). **Lei Feng:** investigation (equal). **Hui Wu:** investigation (equal), methodology (equal). **Tinglei Jiang:** resources (equal). **Jiang Feng:** project administration (equal), resources (equal).

## Conflicts of Interest

The authors declare no conflicts of interest.

## Supporting information


**Figure S1.** The base peak chromatogram (BPC) of six samples detected by LC–MS in satiation state, negative ion on the left, positive ion on the right.
**Figure S2.** The base peak chromatogram (BPC) of six samples detected by LC–MS in sleep state, negative ion on the left, positive ion on the right.
**Figure S3.** The base peak chromatogram (BPC) of six samples detected by LC–MS in fasting state, negative ion on the left, positive ion on the right.
**Figure S4.** The base peak chromatogram (BPC) of six samples detected by LC–MS in activity state, negative ion on the left, positive ion on the right.
**Figure S5.** Box plots of metabolite intensities for 24 samples and QC samples. Rejects are outliers and are not used for subsequent analysis.
**Figure S6.** Principal component analysis (PCA) of all samples.
**Figure S7.** Results of PLS‐DA analysis for the six pairwise comparison groups.
**Figure S8.** Results of OPLS‐DA analysis for the six pairwise comparison groups.
**Figure S9.** Results of response permutation testing (RPT) analysis for the six pairwise comparison groups.
**Figure S10.** The daily dynamic patterns of 145 rhythmic metabolites detected from all 7211 metabolites. These rhythmic metabolites were classified into nine classifications, including lipids and lipid‐like molecules, phenylpropanoids and polyketides, organic acids and derivatives, benzenoids, organic heterocyclic compounds, organic oxygen compounds, nucleosides nucleotides and analogues, organometallic compounds, organooxygen compounds.

**Table S1.** Basic information of all identified metabolites in the liver of Asian particolored bat.

**Table S2.** DAMs detected from six pairwise comparisons.

**Table S3.** KEGG pathways significantly enriched by DAMs detected from six pairwise comparisons, respectively.

**Table S4.** Rhythmic metabolites detected from all 7211 metabolites and 599 DAMs, respectively.

**Table S5.** KEGG pathways significantly enriched by rhythmic metabolites detected from all 7211 metabolites and 599 DAMs, respectively.

**Table S6.** KEGG pathways significantly enriched by DAMs and DEGs from six pairwise comparisons, respectively.

## Data Availability

All relevant data are contained within this research article and in the Supporting Information.
