# Supplementary material for: Comparative Analysis of the Daily Liver Metabolomics of Asian Particolored Bat
Source: Ecol Evol. 2025 Jun 27;15(7):e71666. doi: 10.1002/ece3.71666 (PMC12203122; doi:10.1002/ece3.71666)
Supplement: Supplementary file 1 — Figure S1. The base peak chromatogram (BPC) of six samples detected by LC–MS in satiation state, negative ion on the left, positive ion on the right. Figure S2. The base peak chromatogram (BPC) of six samples detected by LC–MS in sleep state, negative ion on the left, positive ion on the right. Figure S3. The base peak chromatogram (BPC) of six samples detected by LC–MS in fasting state, negative ion on the left, positive ion on the right. Figure S4. The base peak chromatogram (BPC) of six samples detected by LC–MS in activity state, negative ion on the left, positive ion on the right. Figure S5. Box plots of metabolite intensities for 24 samples and QC samples. Rejects are outliers and are not used for subsequent analysis. Figure S6. Principal component analysis (PCA) of all samples. Figure S7. Results of PLS‐DA analysis for the six pairwise comparison groups. Figure S8. Results of OPLS‐DA analysis for the six pairwise comparison groups. Figure S9. Results of response permutation testing (RPT) analysis for the six pairwise comparison groups. Figure S10. The daily dynamic patterns of 145 rhythmic metabolites detected from all 7211 metabolites. These rhythmic metabolites were classified into nine classifications, including lipids and lipid‐like molecules, phenylpropanoids and polyketides, organic acids and derivatives, benzenoids, organic heterocyclic compounds, organic oxygen compounds, nucleosides nucleotides and analogues, organometallic compounds, organooxygen compounds. [file ECE3-15-e71666-s005.pdf]

Comparative analysis of the daily liver metabolomics of Asian particolored bat

Yujia Chu[1], Hui Wang[1]*, Tianhui Wang[1], Jingjing Li[1], Lei Feng[2], Hui Wu[1], Tinglei Jiang[2, 3], and Jiang Feng[1, 2, 3]*

[1] College of Life Science, Jilin Agricultural University, Changchun 130118, China

[2] Jilin Provincial International Cooperation Key Laboratory for Biological Control of Agricultural Pests, Changchun 130118, China

[3] Jilin Provincial Key Laboratory of Animal Resource Conservation and Utilization, Northeast Normal University, Changchun 130117, China

*** Correspondence:**

Hui Wang

wangh681@nenu.edu.cn

Jiang Feng

fengj@nenu.edu.cn

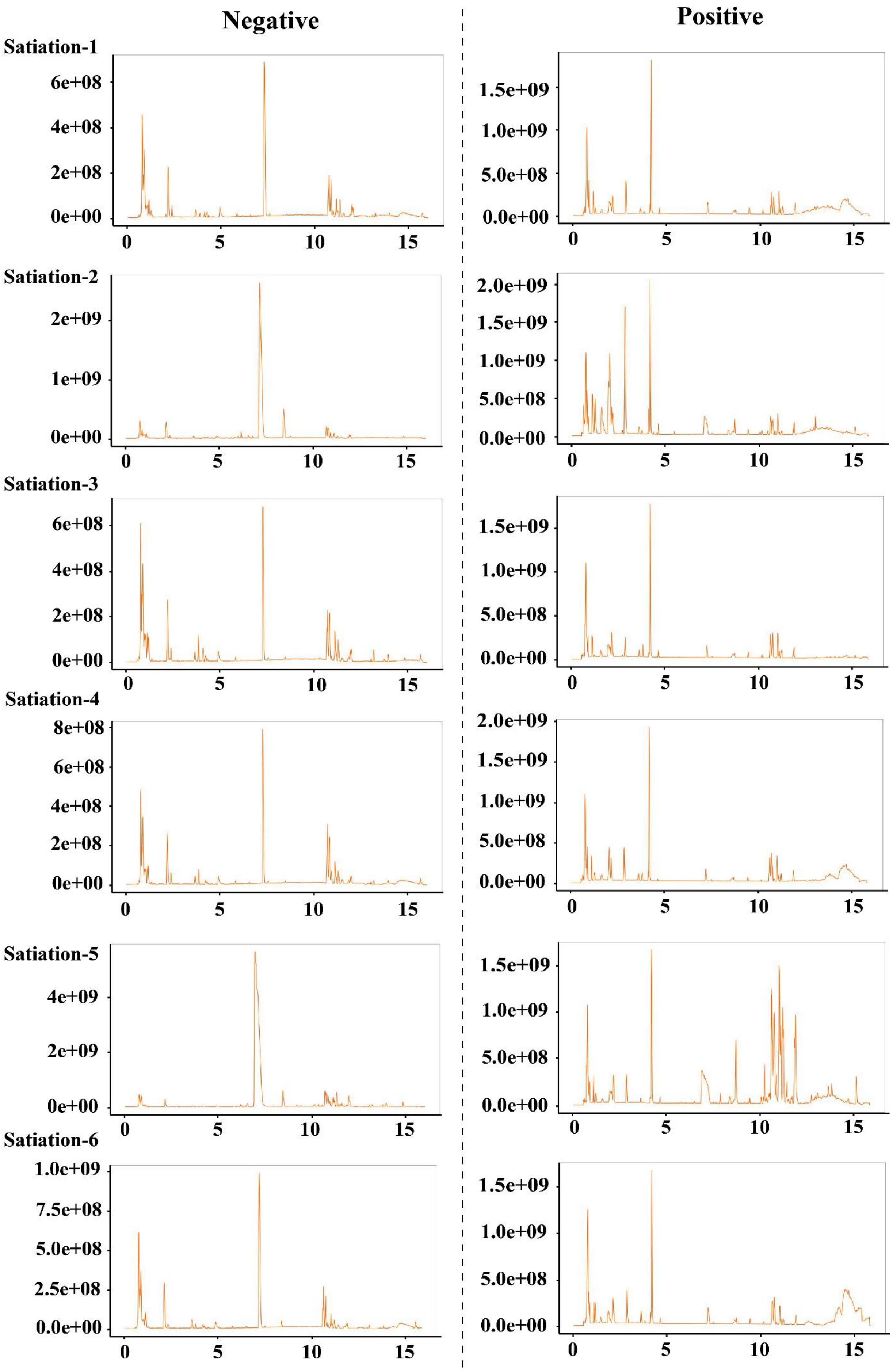

Figure S1. The base peak chromatogram (BPC) of six samples detected by LC-MS in satiation state, negative ion on the left, positive ion on the right.

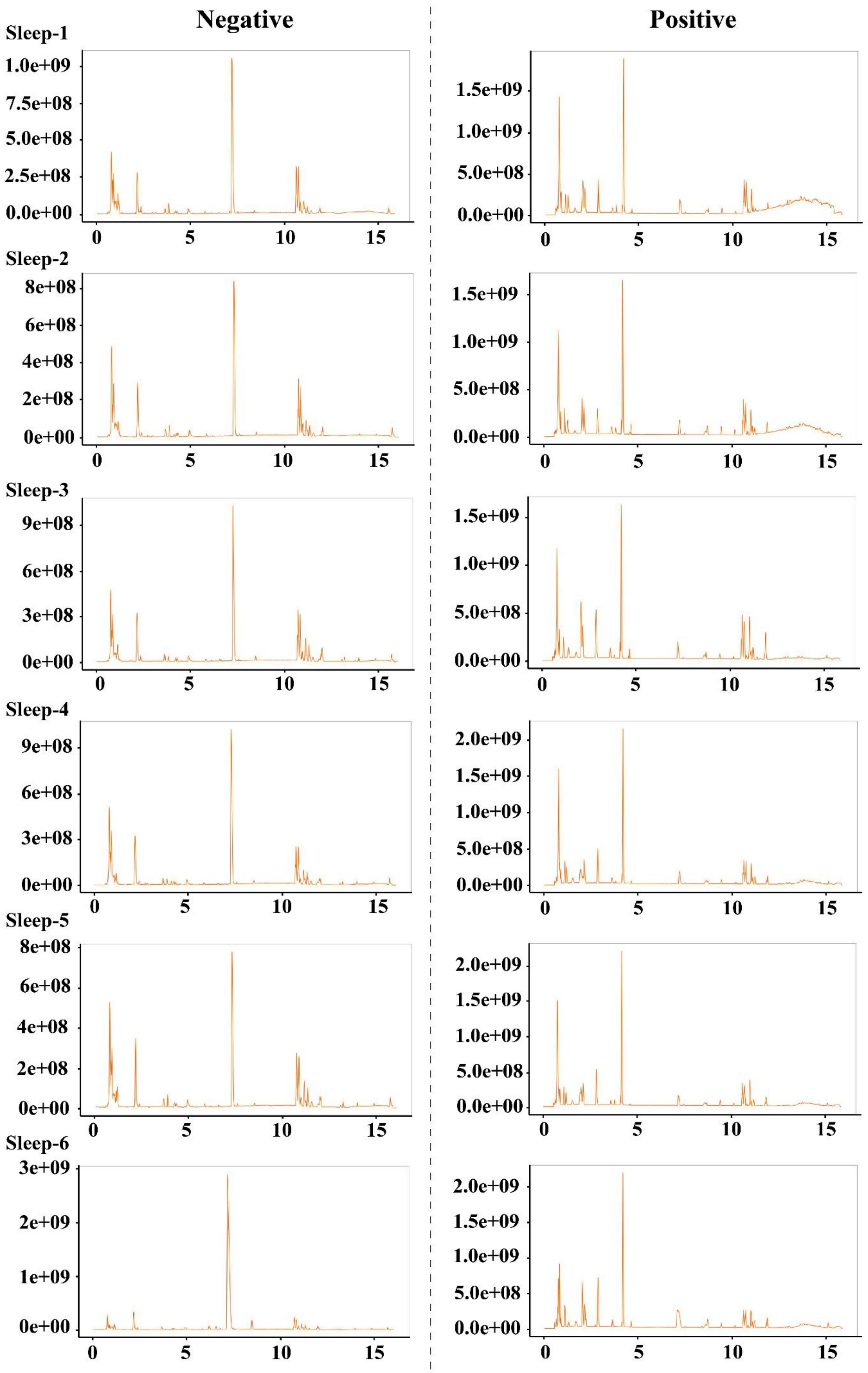

Figure S2. The base peak chromatogram (BPC) of six samples detected by LC-MS in sleep state, negative ion on the left, positive ion on the right.

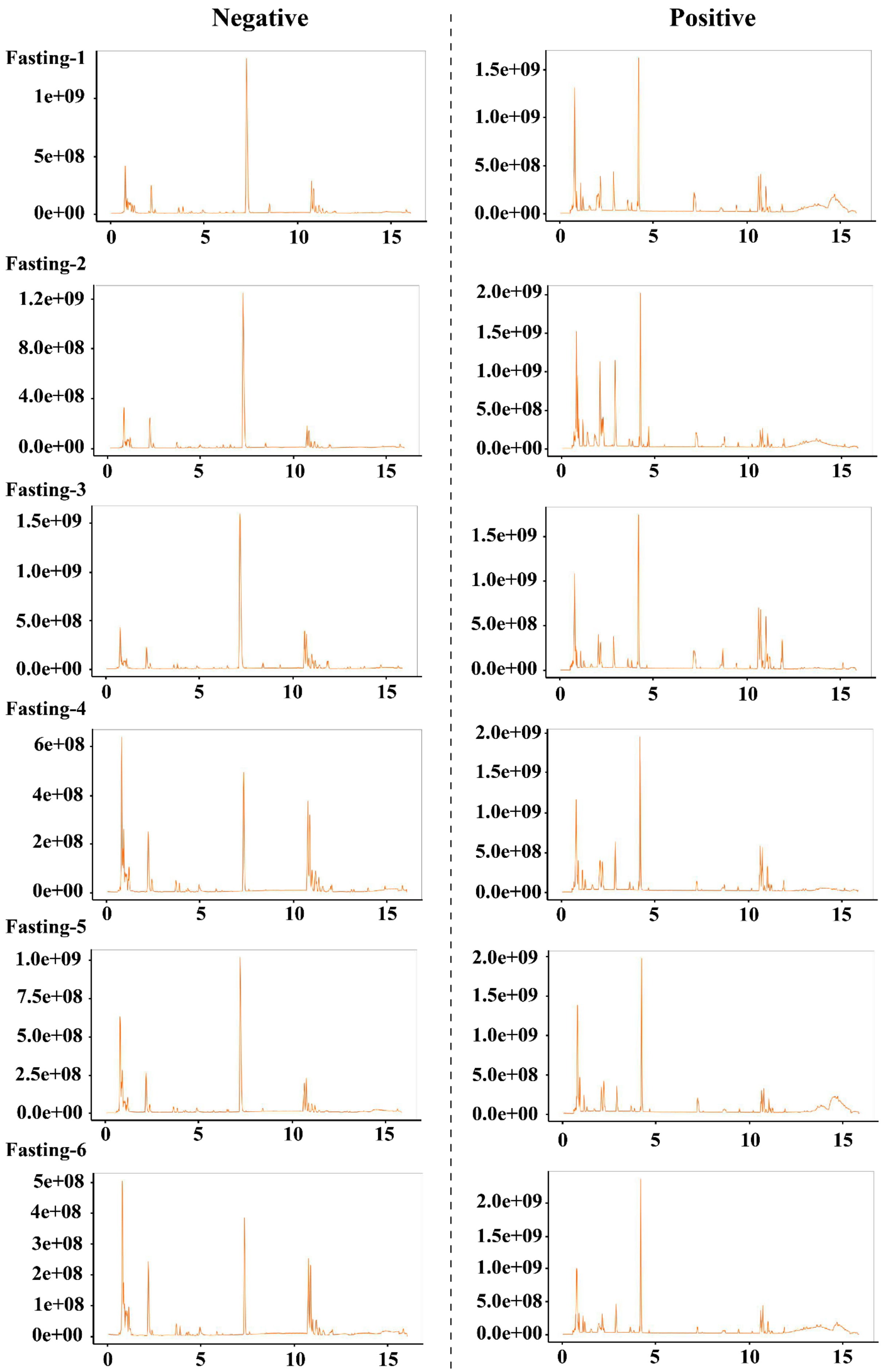

Figure S3. The base peak chromatogram (BPC) of six samples detected by LC-MS in fasting state, negative ion on the left, positive ion on the right.

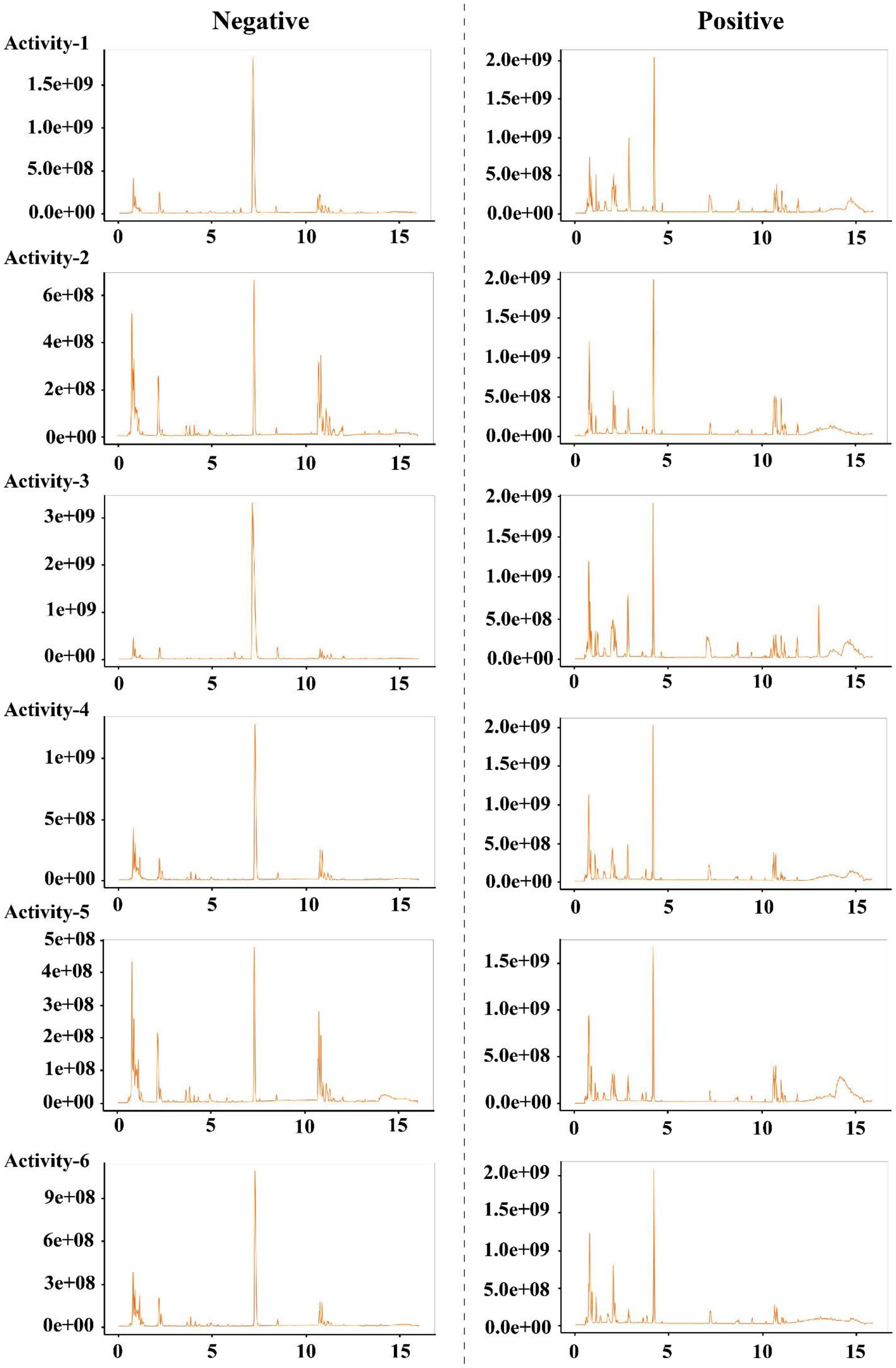

Figure S4. The base peak chromatogram (BPC) of six samples detected by LC-MS in activity state, negative ion on the left, positive ion on the right.

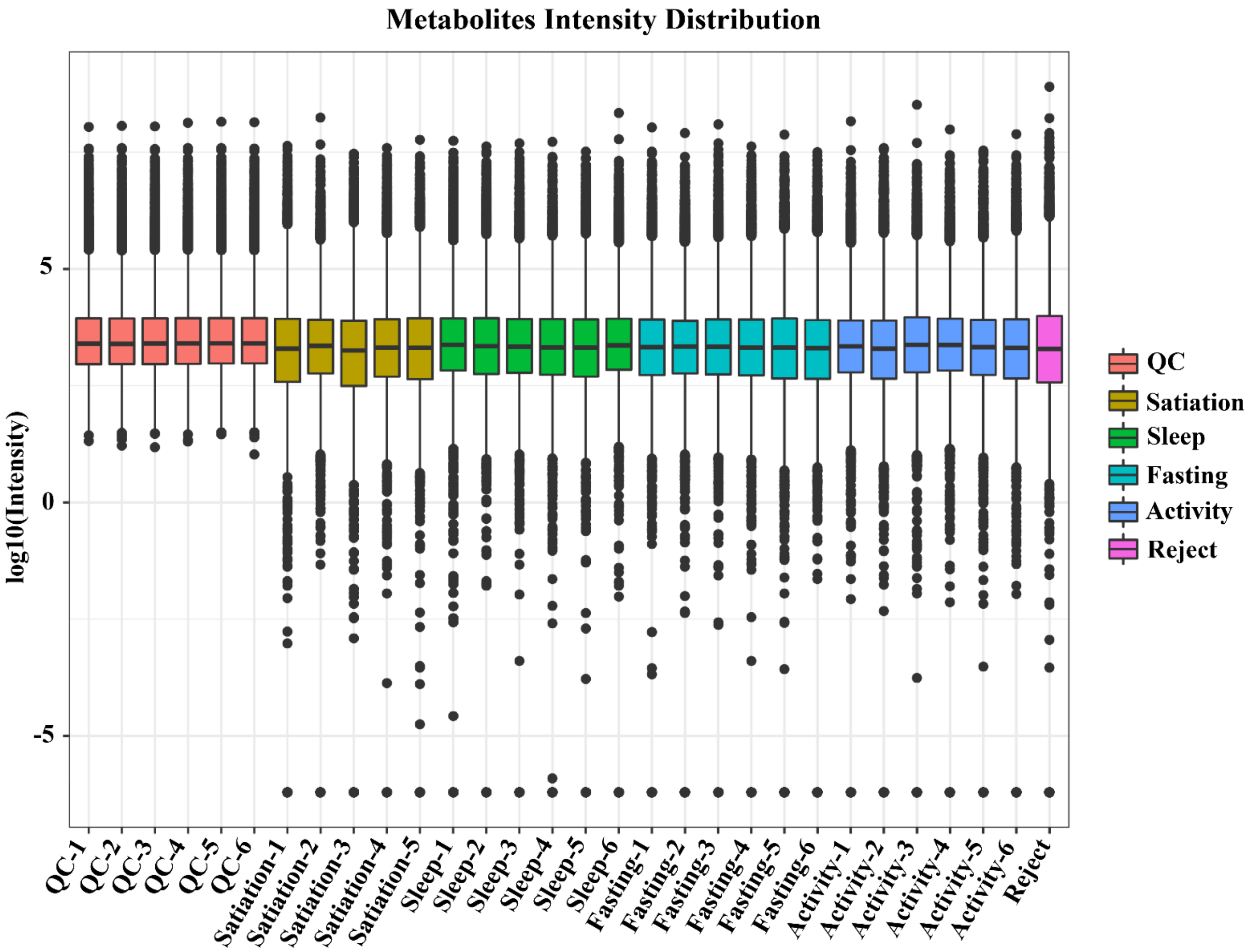

Figure S5. Box plots of metabolite intensities for 24 samples and QC samples. Rejects are outliers and are not used for subsequent analysis.

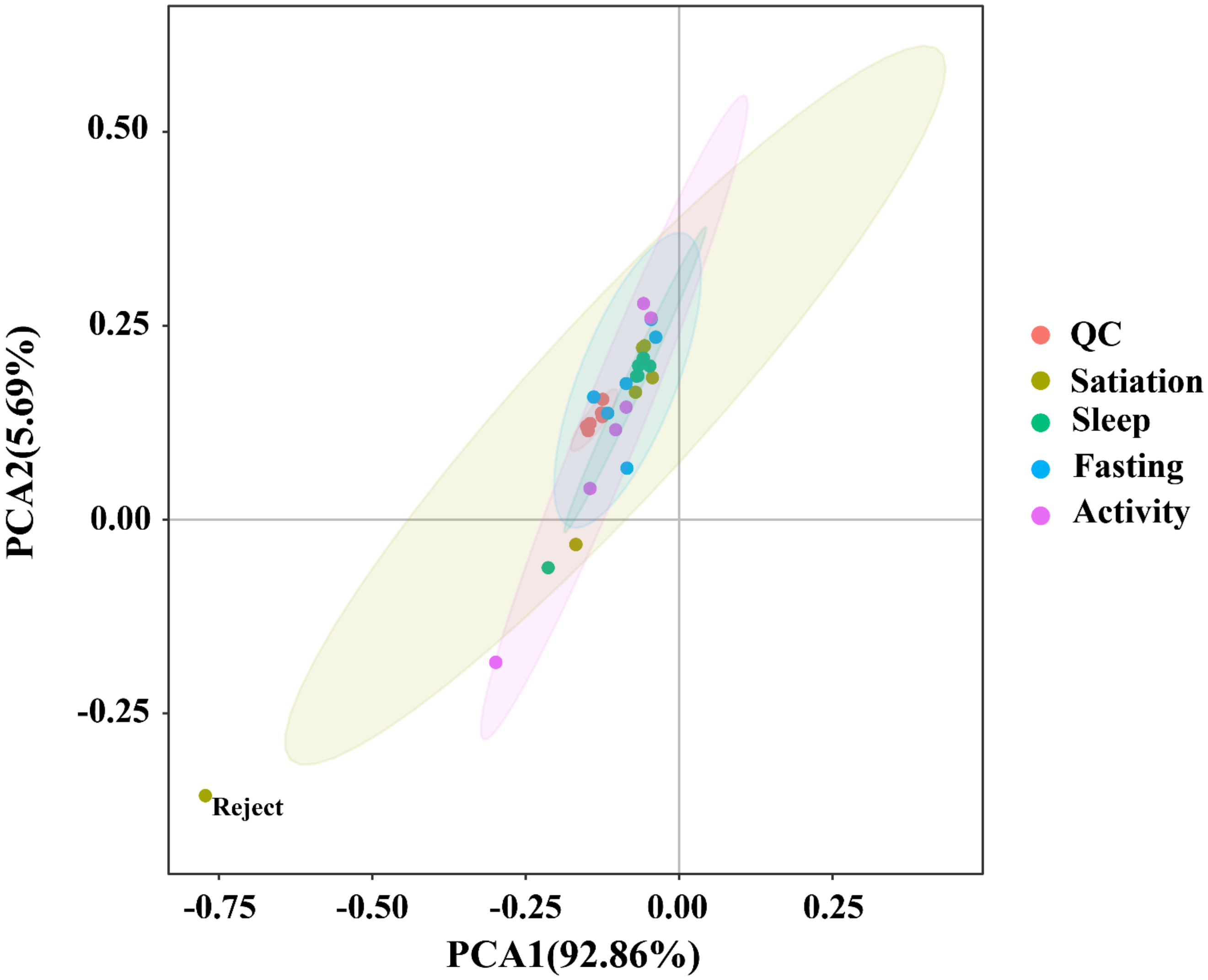

Figure S6. Principal component analysis (PCA) of all samples.

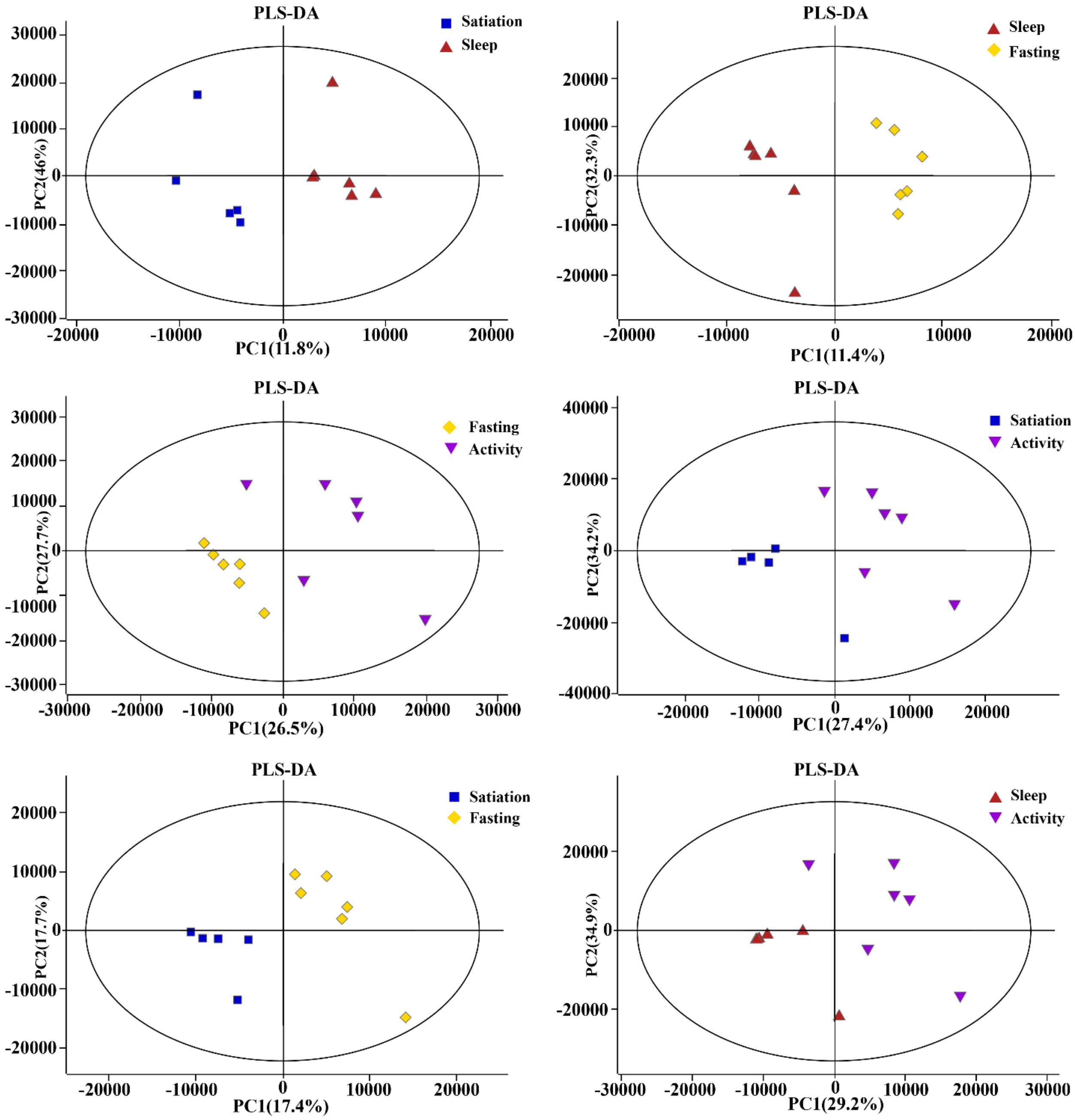

Figure S7. Results of PLS-DA analysis for the six pairwise comparison groups.

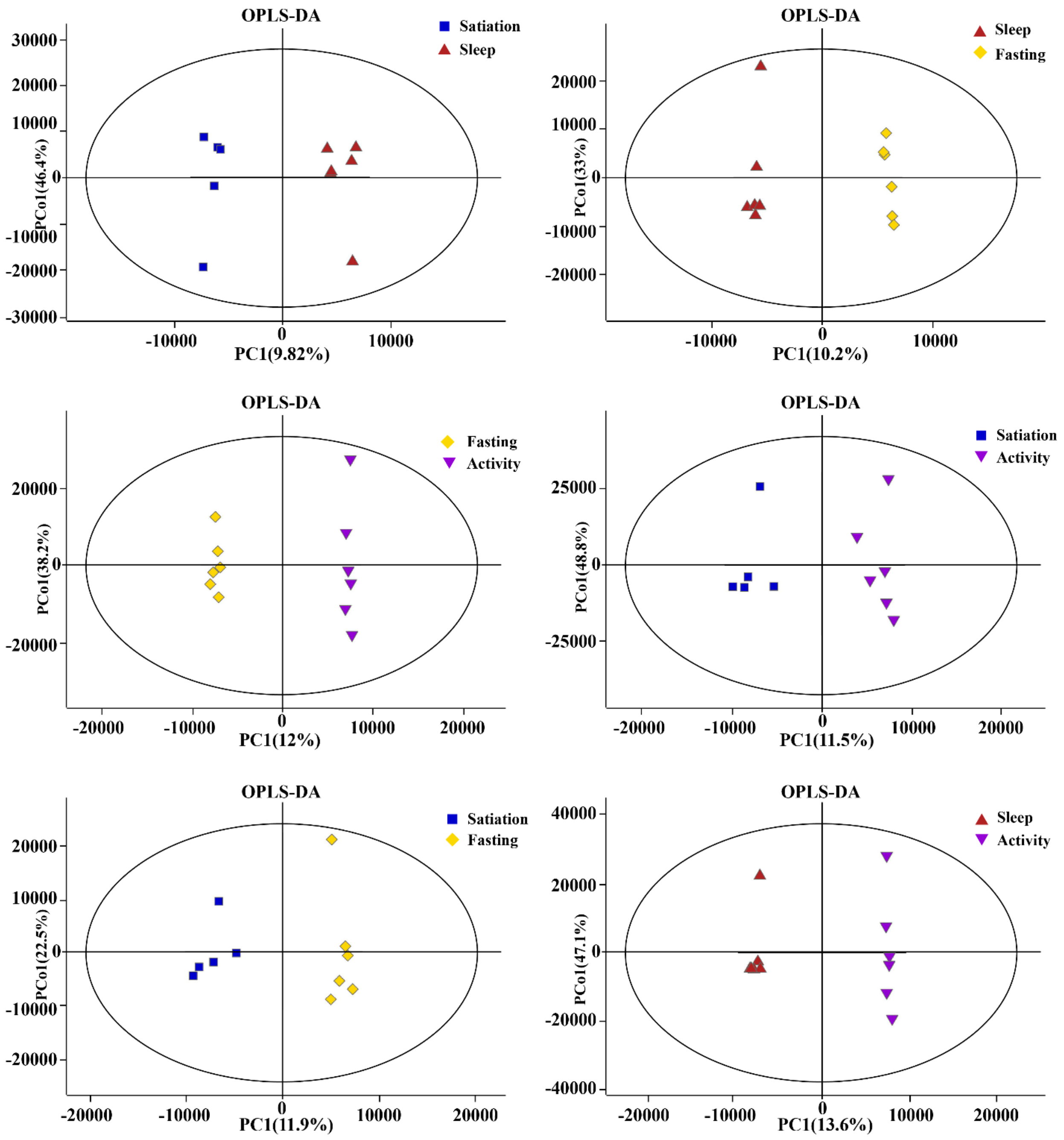

Figure S8. Results of OPLS-DA analysis for the six pairwise comparison groups.

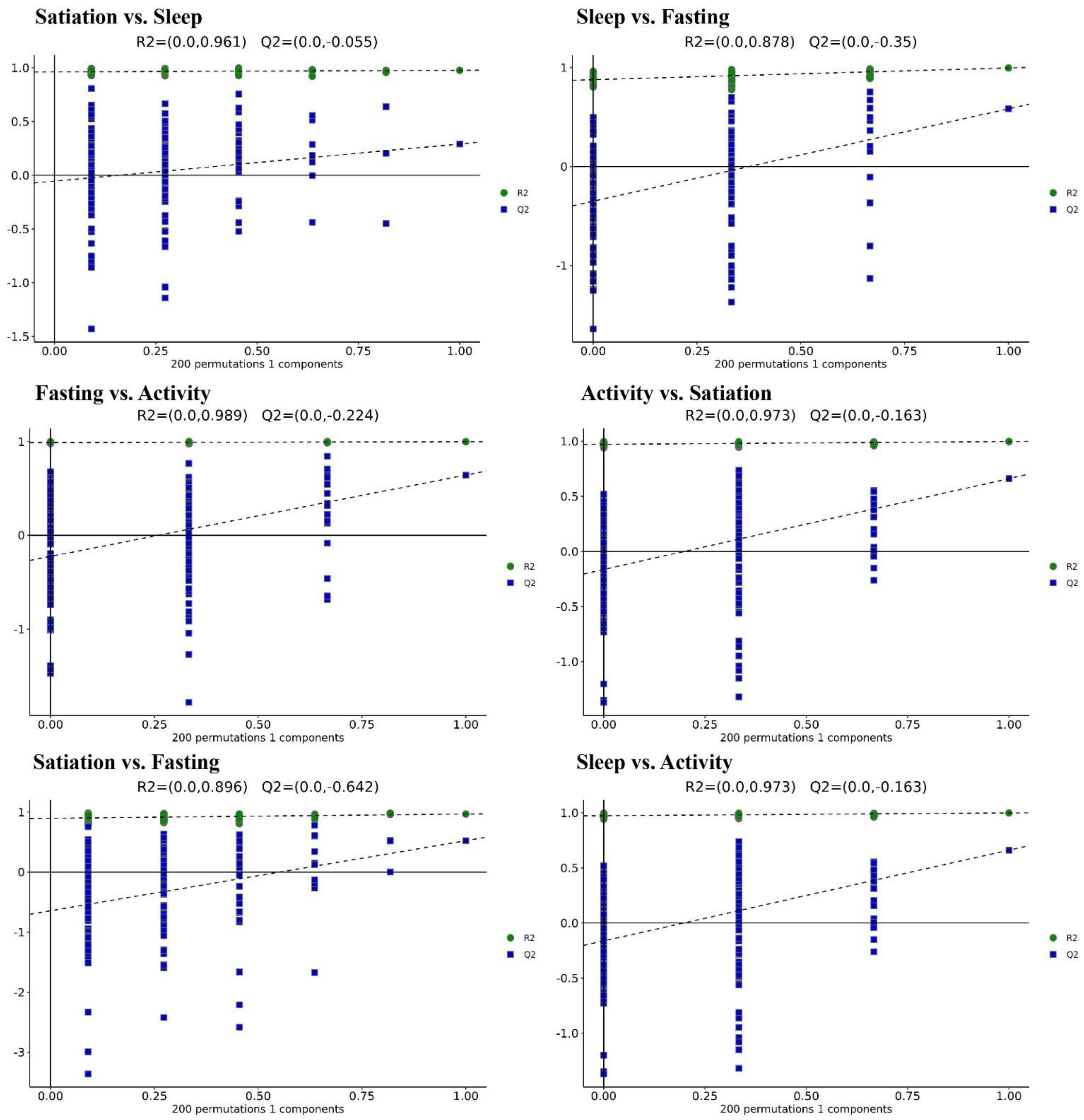

Figure S9. Results of response permutation testing (RPT) analysis for the six pairwise comparison groups.

Lipids and lipid-like molecules

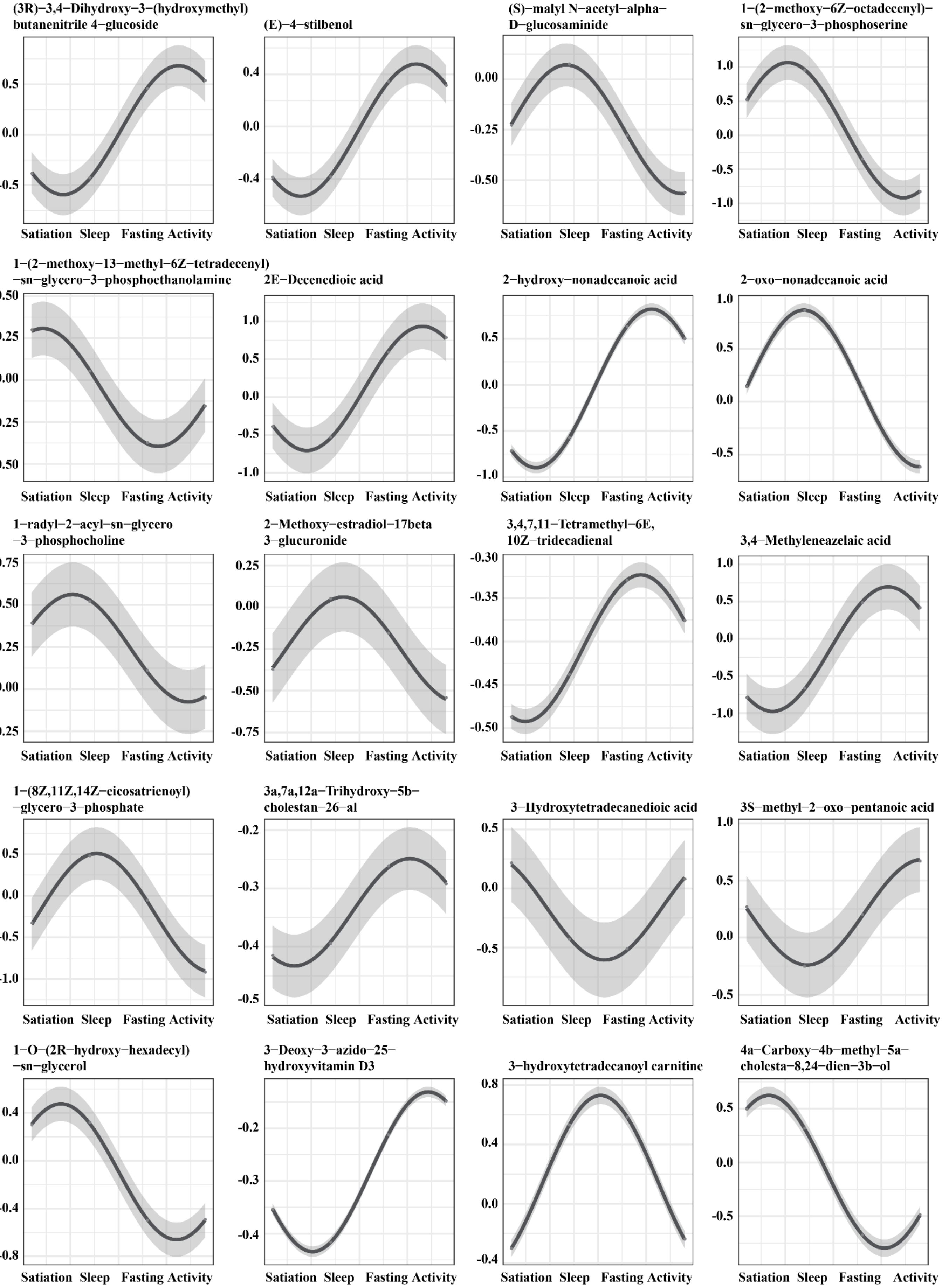

Lipids and lipid-like molecules

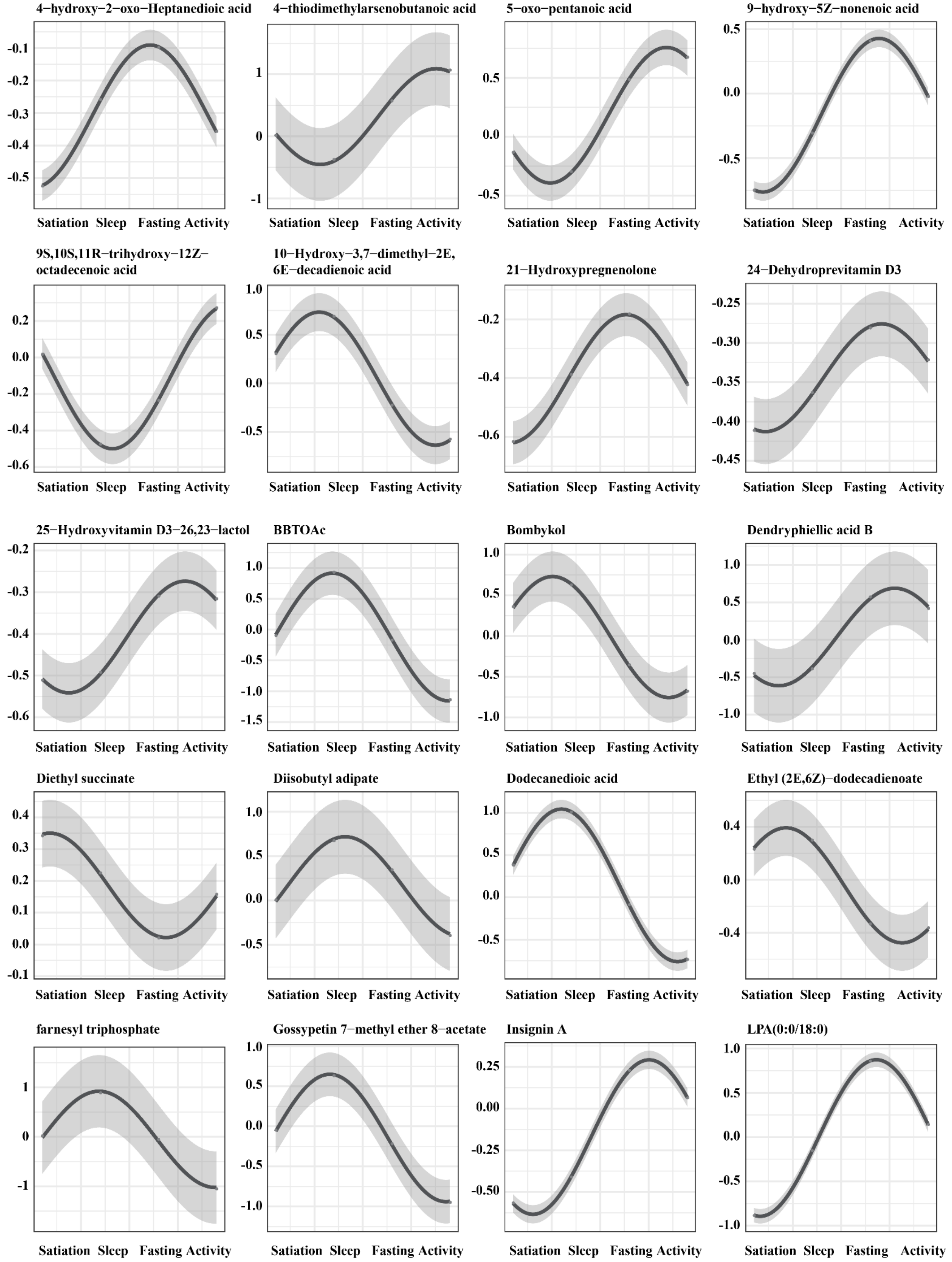

Lipids and lipid-like molecules

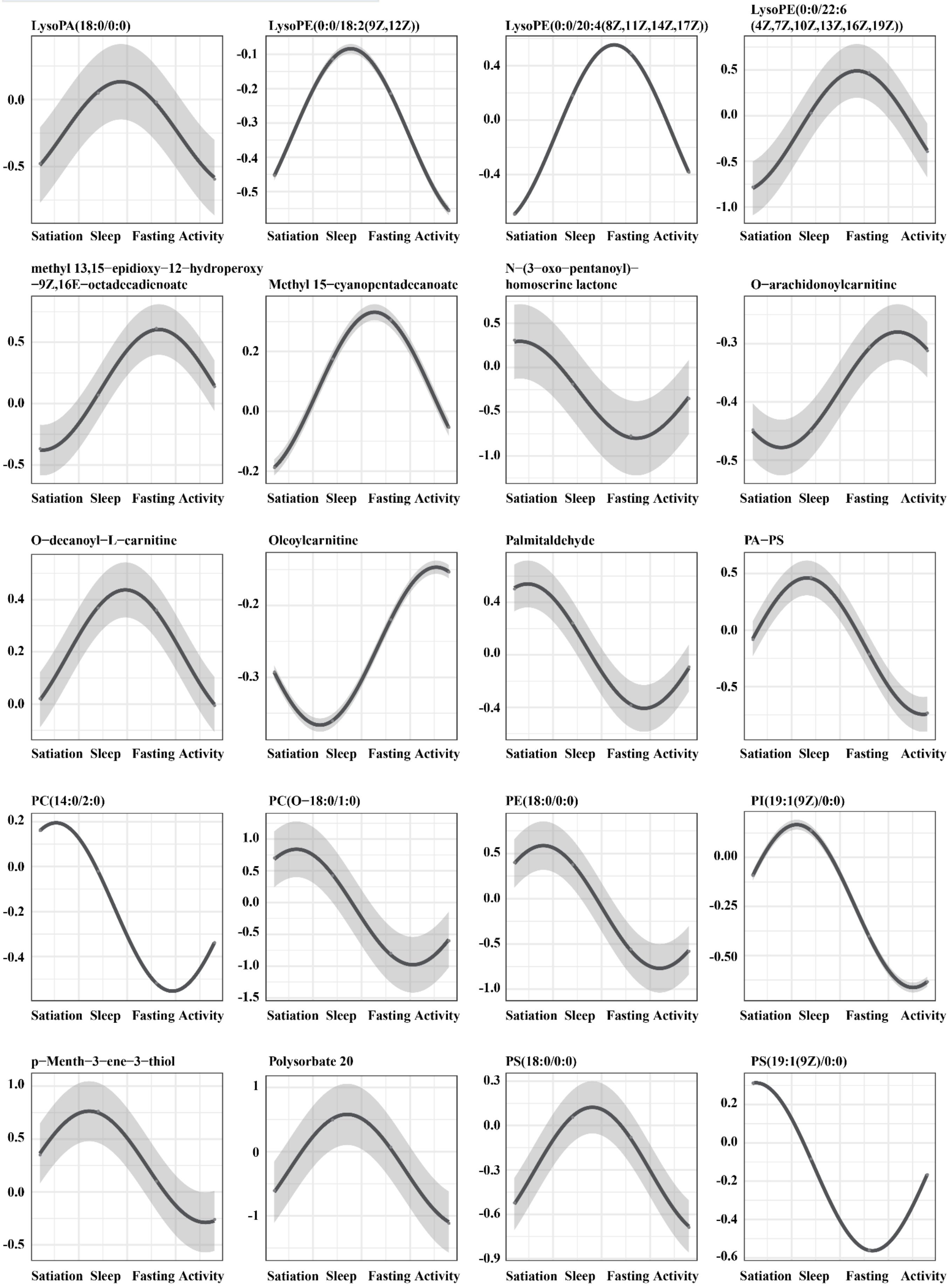

Lipids and lipid-like molecules

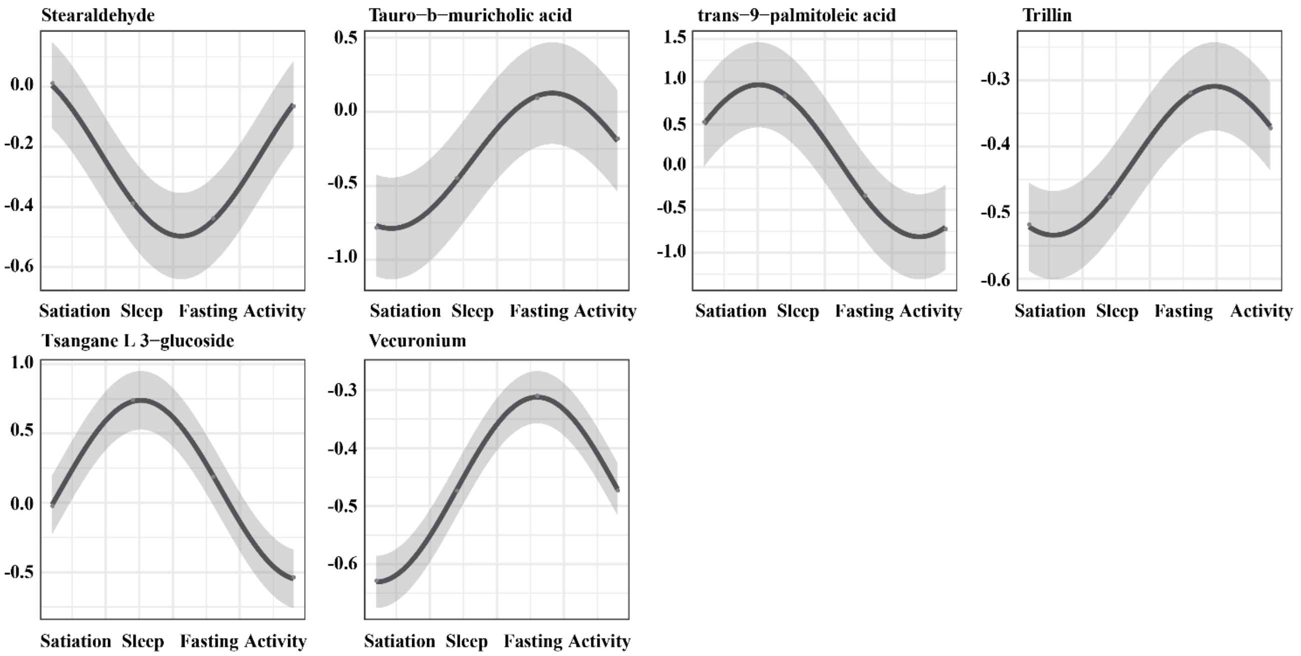

Phenylpropanoids and polyketides

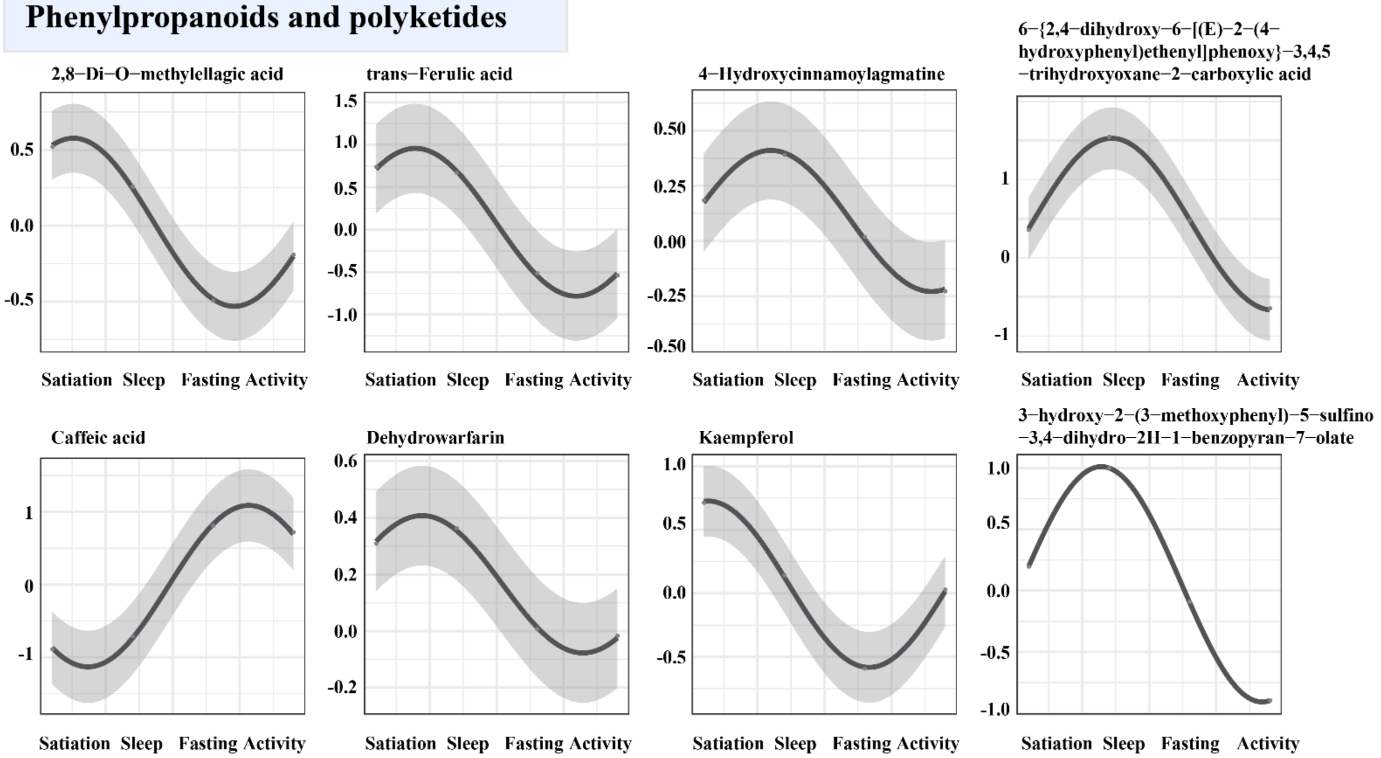

Organic acids and derivatives

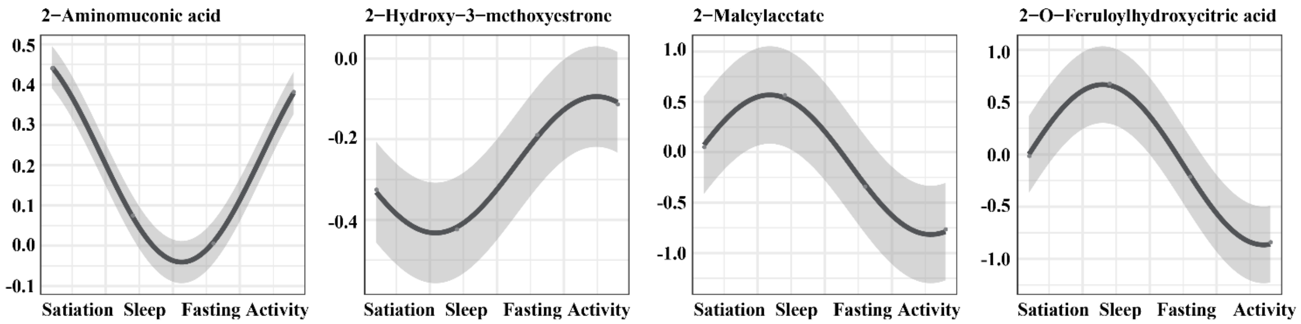

Organic acids and derivatives

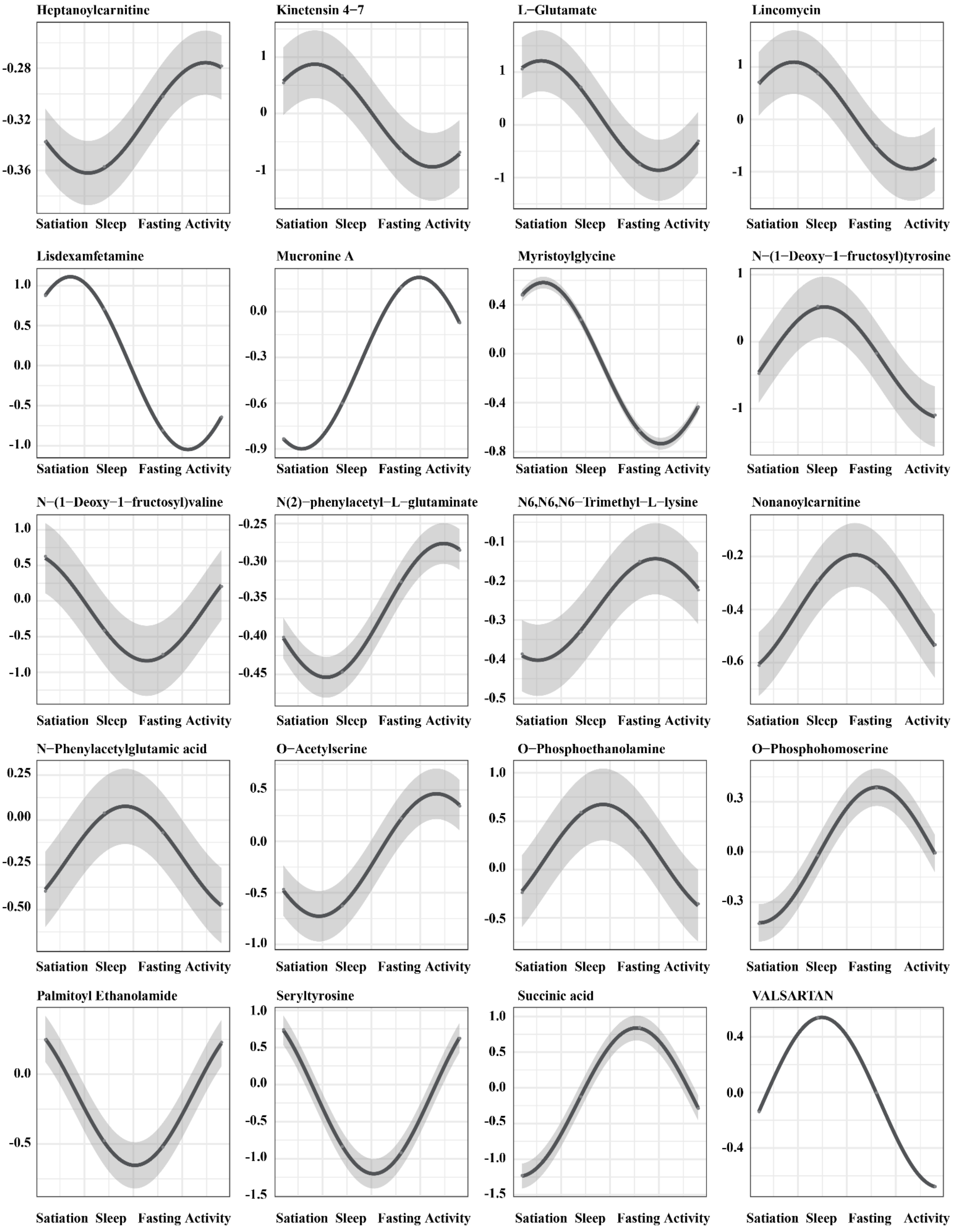

Organic acids and derivatives

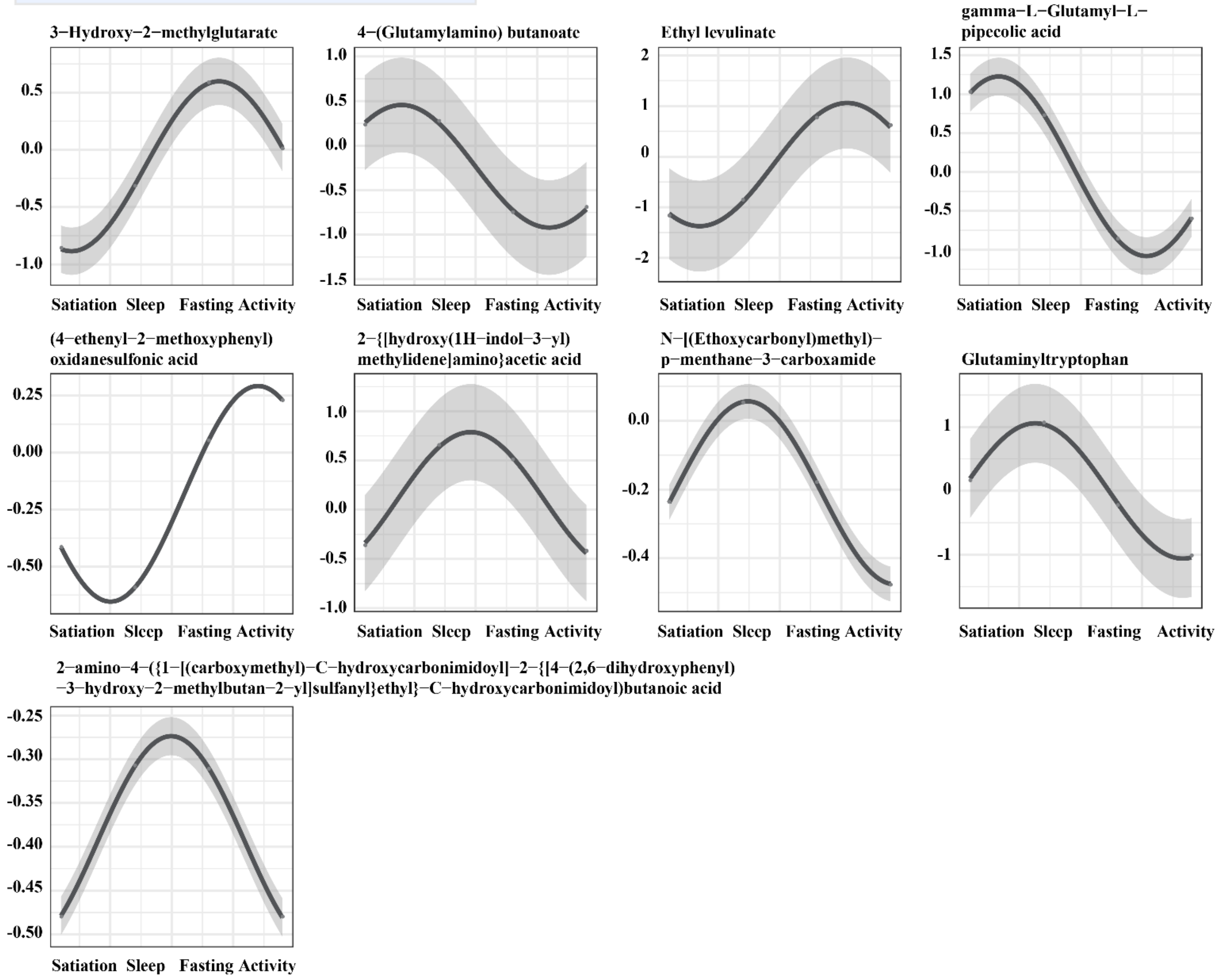

Benzenoids

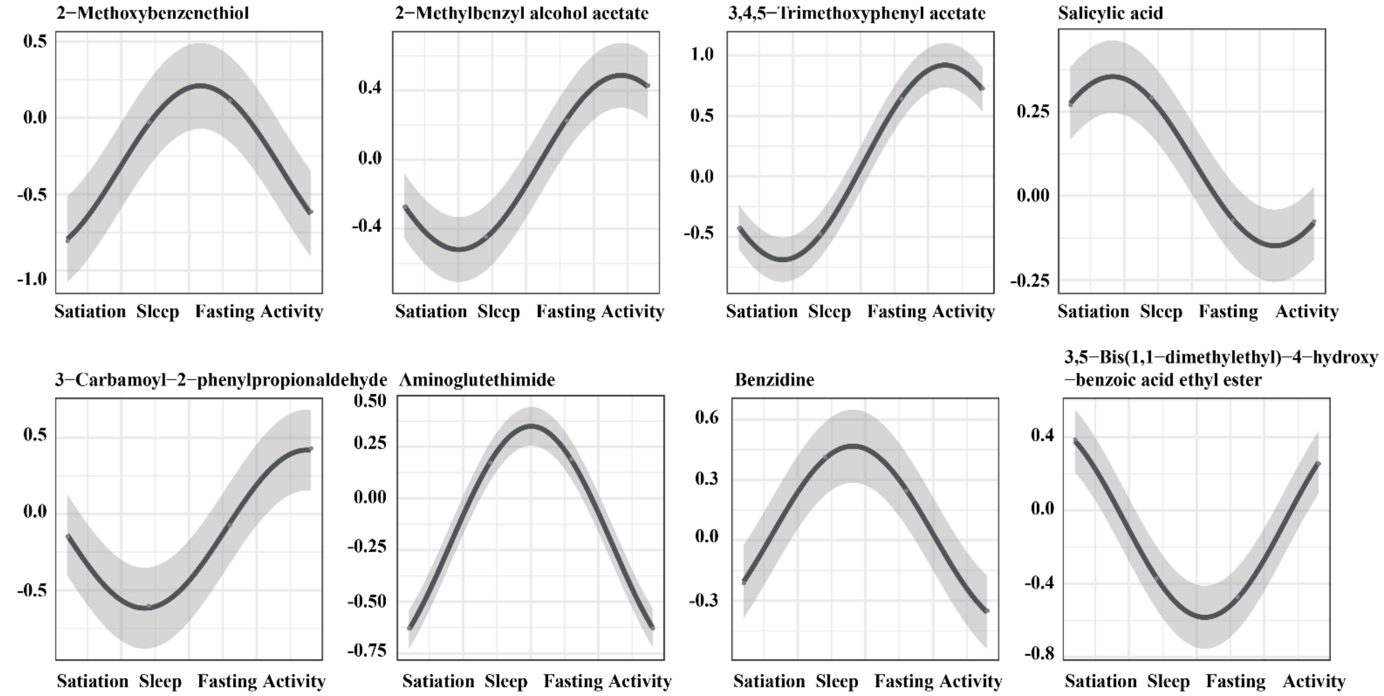

Organoheterocyclic compounds

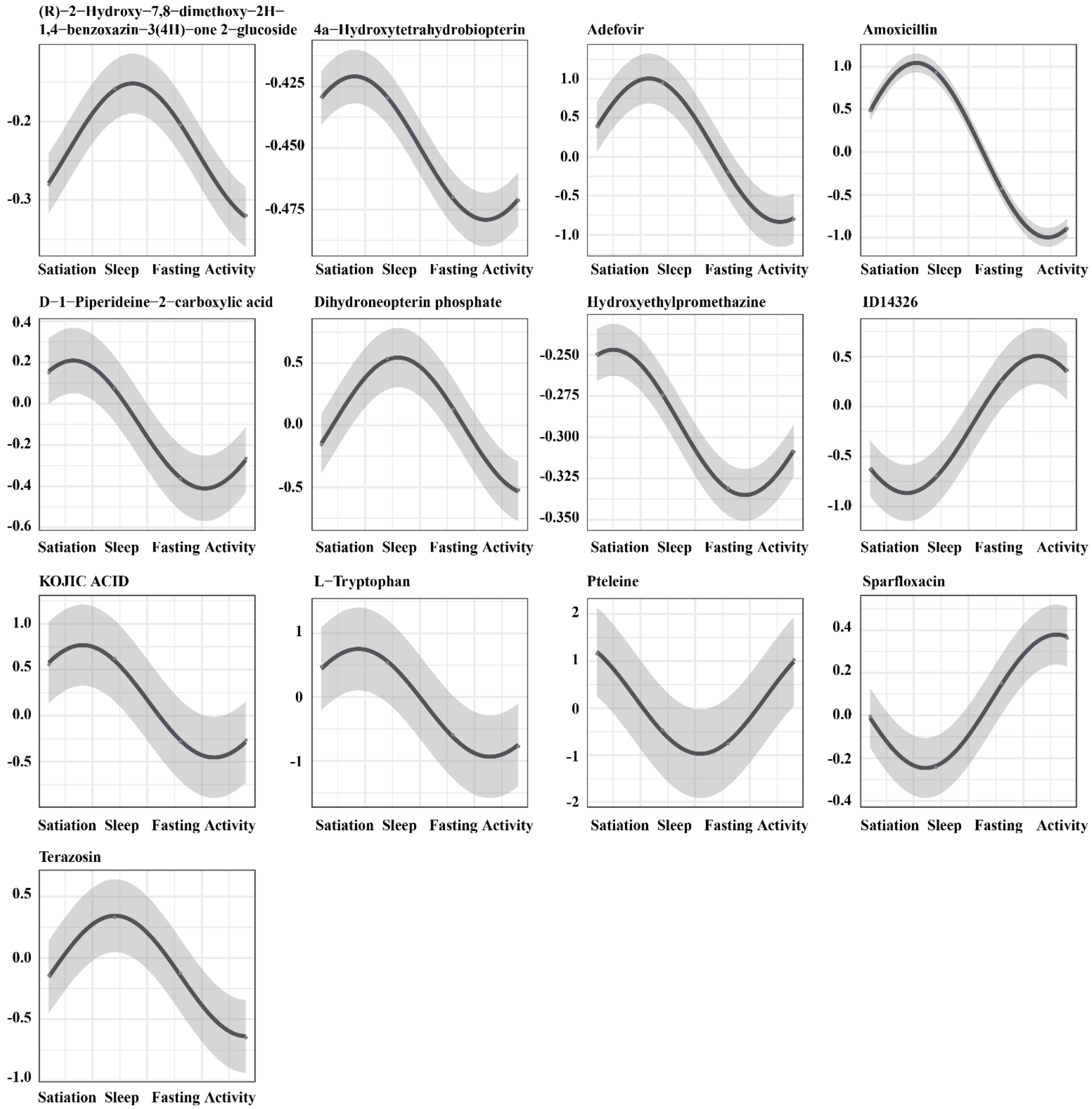

Organic oxygen compounds

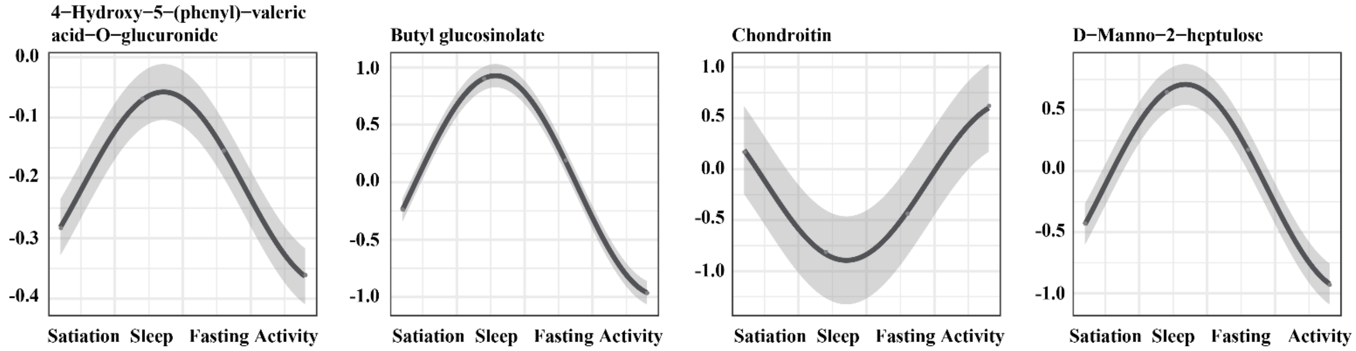

Organic oxygen compounds

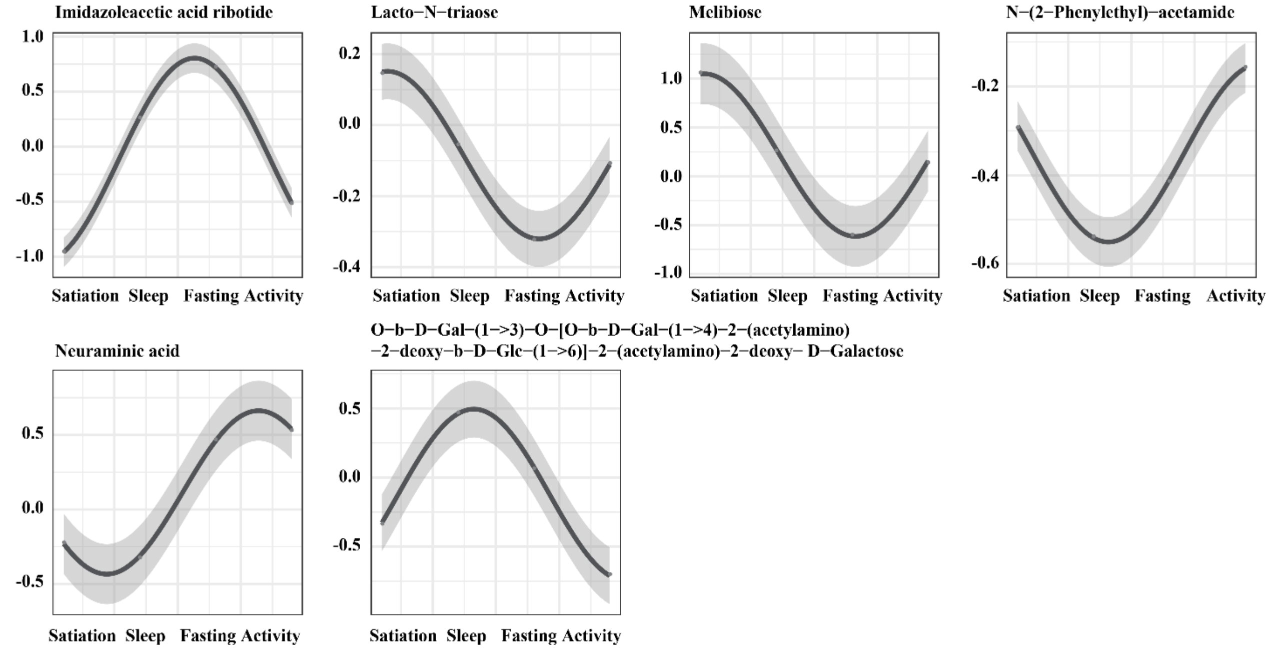

Nucleosides, nucleotides, and analogues

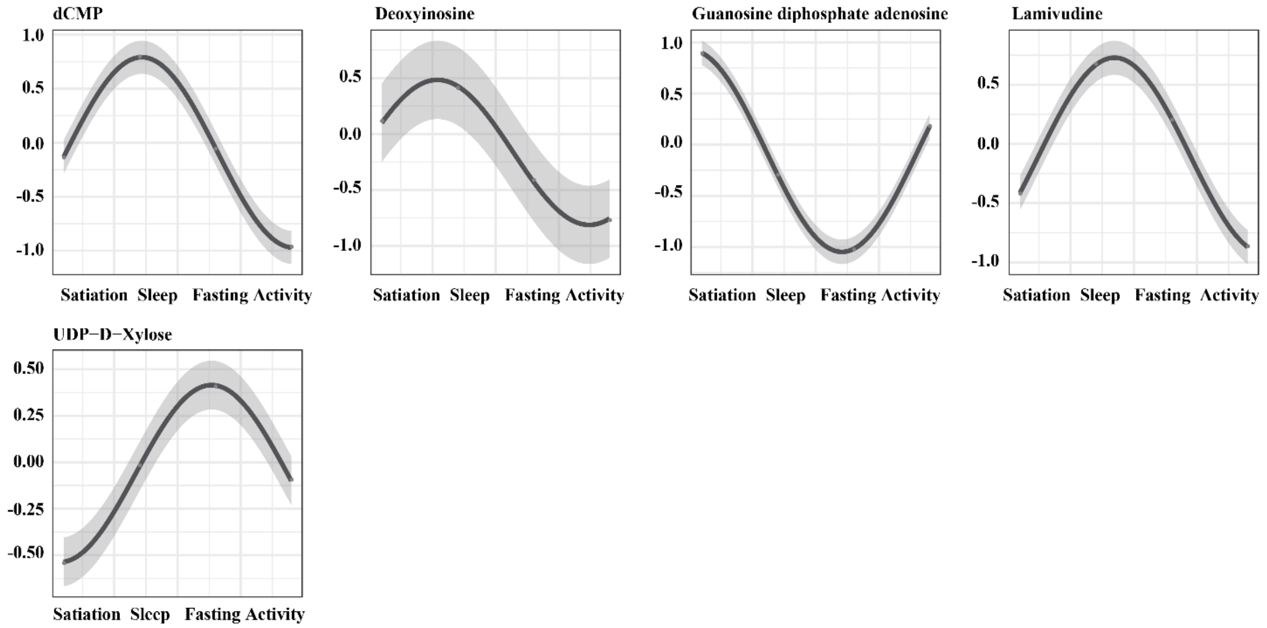

Organometallic compounds

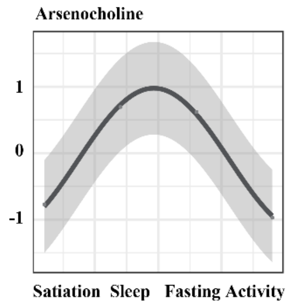

Organooxygen compounds

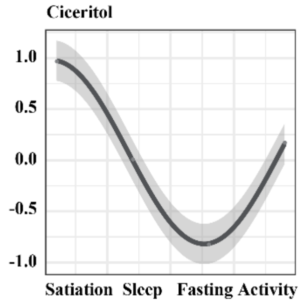

Figure S10. The daily dynamic patterns of 145 rhythmic metabolites detected from all 7211 metabolites. These rhythmic metabolites were classified into nine classifications, including lipids and lipid-like molecules, phenylpropanoids and polyketides, organic acids and derivatives, benzenoids, organic heterocyclic compounds, organic oxygen compounds, nucleosides nucleotides and analogues, organometallic compounds, organooxygen compounds.
